# Strict conformational demands of RNA cleavage in bulge-loops created by peptidyl-oligonucleotide conjugates

**DOI:** 10.1093/nar/gkaa780

**Published:** 2020-10-03

**Authors:** Yaroslav Staroseletz, Bahareh Amirloo, Aled Williams, Alexander Lomzov, Kepa K Burusco, David J Clarke, Tom Brown, Marina A Zenkova, Elena V Bichenkova

**Affiliations:** Institute of Chemical Biology and Fundamental Medicine SB RAS, 8 Laurentiev Avenue, 630090 Novosibirsk, Russia; School of Health Sciences, Faculty of Biology, Medicine and Health, University of Manchester, Oxford Road, Manchester M13 9PT, UK; School of Health Sciences, Faculty of Biology, Medicine and Health, University of Manchester, Oxford Road, Manchester M13 9PT, UK; Institute of Chemical Biology and Fundamental Medicine SB RAS, 8 Laurentiev Avenue, 630090 Novosibirsk, Russia; School of Health Sciences, Faculty of Biology, Medicine and Health, University of Manchester, Oxford Road, Manchester M13 9PT, UK; School of Health Sciences, Faculty of Biology, Medicine and Health, University of Manchester, Oxford Road, Manchester M13 9PT, UK; Department of Chemistry, Chemistry Research Laboratory, University of Oxford, 12 Mansfield Road, Oxford, OX1 3TA, UK; Institute of Chemical Biology and Fundamental Medicine SB RAS, 8 Laurentiev Avenue, 630090 Novosibirsk, Russia; School of Health Sciences, Faculty of Biology, Medicine and Health, University of Manchester, Oxford Road, Manchester M13 9PT, UK

## Abstract

Potent knockdown of pathogenic RNA *in vivo* is an urgent health need unmet by both small-molecule and biologic drugs. ‘Smart’ supramolecular assembly of catalysts offers precise recognition and potent destruction of targeted RNA, hitherto not found in nature. Peptidyl-oligonucleotide ribonucleases are here chemically engineered to create and attack bulge-loop regions upon hybridization to target RNA. Catalytic peptide was incorporated either via a centrally modified nucleotide (Type 1) or through an abasic sugar residue (Type 2) within the RNA-recognition motif to reveal striking differences in biological performance and strict structural demands of ribonuclease activity. None of the Type 1 conjugates were catalytically active, whereas all Type 2 conjugates cleaved RNA target in a sequence-specific manner, with up to 90% cleavage from 5-nt bulge-loops (BC5-α and BC5L-β anomers) through multiple cuts, including in folds nearby. Molecular dynamics simulations provided structural explanation of accessibility of the RNA cleavage sites to the peptide with adoption of an *‘in-line’* attack conformation for catalysis. Hybridization assays and enzymatic probing with RNases illuminated how RNA binding specificity and dissociation after cleavage can be balanced to permit turnover of the catalytic reaction. This is an essential requirement for inactivation of multiple copies of disease-associated RNA and therapeutic efficacy.

## INTRODUCTION

Irreversible sequence-selective cleavage of disease-associated RNA is recognized as a promising therapeutic strategy against abnormal gene expression in disease states (1–3). In contrast to low molecular weight drugs, which are designed to confront disease *downstream*, when it is already established in cell components at the level of expressed pathogenic proteins, *upstream* RNA targeting allows a desirable therapeutic response through controlled translational arrest of such proteins, well before they are translated into disease states. Contrary to small-molecule drug chemotherapeutic treatments, which suffer from cytotoxicity, with inevitable side effects which limit their success, (4,5) RNA therapeutics offer the opportunity of high selectivity, improved potency and reduced toxicity (6). The lack of repair mechanisms and accessibility of many RNAs in the cytoplasm make RNA an attractive and less risky biotarget than DNA, because RNA targeting avoids gene manipulation or editing (*cf*. with CRISPR). RNA-targeting interventions can be directed toward a diverse variety of RNA sequences ranging from messenger RNAs encoding pathogenic proteins, or viral genomic RNAs, to non-coding RNAs involved in cellular signaling pathways. It is now well recognized that non-coding RNA molecules (e.g. miRNA, lncRNA and piRNA) play a major role in numerous human diseases including ovarian, breast and lung cancer, as well as in inflammatory and degenerative diseases (7–9). Currently, RNA degradation and protein expression knockdown by siRNA and antisense oligonucleotide (ASO) therapeutics must recruit enzyme complexes: RNA-induced silencing complex (RISC) and ribonuclease H, respectively. However, recruiting enzymes, which are required to regulate normal cellular RNA, inevitably led to toxic effects such as upregulation of oncogenes (e.g. HMGA2, CCND1 and DUSP2) (10). Also, the use of siRNA technology often triggers the activation of the innate immune response, (11,12) which presents another clinical barrier. ASOs struggle with their 1:1 binding stoichiometry, both to reach their therapeutic threshold and to maintain a therapeutic effect against fresh transcription and turnover (3). This requires high concentrations of ASOs to be maintained within the cellular environment for lengthy periods, which is often difficult to achieve due to their rapid clearance and poor cellular delivery, even if insufficient chemical stability of oligonucleotide therapeutics is overcome (13). Therefore, the development of novel chemical agents capable of achieving potent, irreversible destruction of disease-relevant RNA sequences without reliance on intracellular molecular components is of particular importance.

Chemically engineered artificial (or chemical) ribonucleases (14–20) offer unique opportunities for catalytic destruction of disease-relevant RNA without engaging complex multi-component machinery (e.g. RISC, RNase H) that normally act via recruitment of a guide RNA or DNA sequence for target recognition and a *protein–RNA* assembly for RNA cleavage. We have recently developed peptidyl-oligonucleotide conjugates (15–20) (POCs) as a new class of metal-free chemical ribonucleases, which consist of an antisense oligonucleotide targeted toward a specific RNA sequence, and an RNA-cleaving catalyst (e.g. amphiphilic peptide). Such bioconjugates (15–19) are capable of inducing complete phosphodiester transesterification of target RNA sequence without requirement of enzymes, additional endogenous cofactors or exogenous agents (e.g. metal ions) that can fluctuate within and between cell types. Recently, we have demonstrated (17,20) that this type of biologically active conjugates can selectively knockdown highly oncogenic microRNA (miR-21) by irreversible cleavage and thereby induce apoptosis in tumor cells, suppress their invasive properties and knockdown tumor proliferation *in vitro*. A single treatment of tumor cells with such catalytic conjugates eliminated their malignant behavior and persisted to retard subsequent tumor growth *in vivo* after their transplantation into mice (20).

In order to improve their potency against disease-relevant RNAs, so that dosage and systemic delivery issues are overcome, it is imperative to ensure that each POC molecule inactivates many copies of pathogenic RNA for a lengthy period of time. This can only be achieved through catalytic turnover which persists in cells. Reaction turnover of multiple RNA substrates requires rapid release of cleaved RNA fragments after each cleavage event, to liberate the POC from the hybridized complex and allow it to attack the next RNA molecule. However, the high stability of hybridized *RNA:DNA* heteroduplexes, hitherto essential to ensure the high level of precision in RNA recognition to avoid off-target effects and associated toxicity, opposes the ability of conjugates to leave RNA after cleavage (14,15). Although our ‘hairpin’ peptidyl-oligonucleotide conjugates have recently demonstrated the ability to cleave RNA in a multiple turnover mode by working synergistically with cellular enzymes (20), they continue to rely on the variable cytosolic abundance of RNase H1 (21) to achieve turnover of RNA substrates. The main challenge is therefore to develop fully independent, self-reliant RNA-specific conjugates that combine absolute sequence-specificity with a high level of catalytic turnover, which can operate autonomously under physiological conditions. This can be achieved by optimizing the length of the conjugate recognition motifs and the point for attachment of the catalytic functionality to ensure that the binding affinity of the RNA cleavage products toward the conjugate is considerably lower than that of the intact RNA chain (14). Artificial ribonucleases with metal-coordinated (e.g. Dy(III), Cu(II), Eu(III)) RNA-cleaving constructs, although toxic and unstable, provide evidence that, in principle, catalytic turnover of RNA substrates can be achieved (22–26). However, they are susceptible to metal loss from their coordinating ligands and may cause degradation of non-target biopolymers, (14–15,27) thus leading to undesirable side effects and cytotoxicity. Our focus is therefore to develop *metal-independent* ribonucleases, suitable for *in vivo* studies and therapeutic applications.

The nature of the RNA target is particularly important when designing artificial ribonucleases. Single-stranded RNA regions that can be found in bulge-loops are more susceptible to cleavage than duplex RNA fragments (27,28). This is due to the orientation of the 2′- hydroxyl in an RNA duplex being unsuitable for intramolecular transesterification, and a conformational change would require unfavorable duplex disruption (29). Cleavage within RNA bulge-loops is widely regarded as a method to encourage catalytic turnover of chemical nucleases (30,31). In order to induce bulge-loops in a target RNA, the sequence of the conjugate recognition motif must be designed so that a short region (2–5nt) in the middle of the target RNA sequence remains non-complementary, thus forcing the RNA to adopt a bulge-loop structure upon hybridization. The size of the bulge-loop can be fine-tuned by altering the size of the non-complementary regions. Previously, 3- and 4-member bulge-loops have shown the greatest potential for cleavage (30,31) by different catalysts, presumably due to their ability to provide the optimum flexibility for the *‘in-line’* attack of the 2′-oxyanion onto the bridging phosphodiester group, the rate-limiting step of the phosphodiester cleavage mechanism (30,31). Recently developed bulge-loop inducing peptide nucleic acid-based conjugates, PNAzymes, carrying toxic Cu^2+^-2,9-dimethylphenanthroline or tris(2-aminobenzimidazole) cleaving units demonstrated multiple substrate turnover against a model 15-nt RNA (23,32,33).

**Figure 1.**
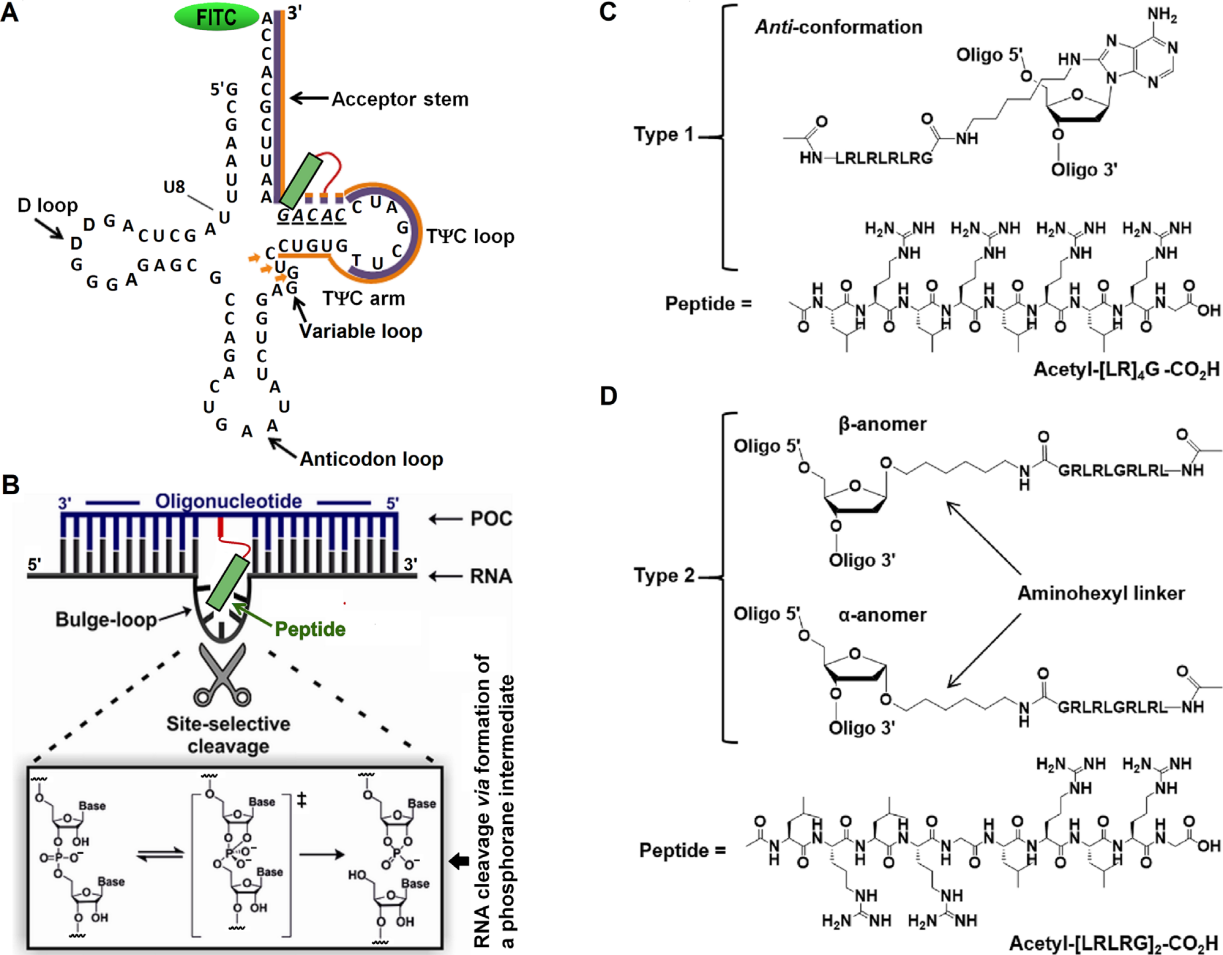
(**A**) Secondary structure of *in vitro* transcript of tRNA^Phe^ showing the TΨC arm and acceptor stem binding sites targeted by the recognition motifs of the Type 1 and Type 2 conjugates (shown by purple and orange lines, respectively). Solid lines represent regions common for all conjugates from each series. Orange arrows indicate the extended binding region for the elongated conjugates BC5L-α and BC5L-β. Dotted lines show variable regions between oligonucleotide sequences bearing the catalytic peptide (green). The bulge-loop forming target site is underlined in italics. (**B**) Illustration of the bulge-loop inducing peptidyl-oligonucleotide conjugate (POC, shown here by blue line) bound to the target RNA sequence incorporating the 3′ acceptor stem and TΨC arm of tRNA^Phe^ (RNA, shown here by black line). Non-complementary regions around the peptide create a bulge-loop in the target RNA, which is susceptible to cleavage by the catalytic peptide. (**C** and**D**) Structural features of the peptide conjugation points for Type 1 and Type 2 bulge-loop inducing conjugates. (**C**) Type 1 conjugates (BC2–BC5) incorporate acetyl-[LR]_4_G-CO_2_H peptide via an internal aminohexyl-modified adenosine, here shown in more favored *anti* conformation. (**D**) Type 2 conjugates (BC2-α-BC5L-α and BC2-β-BC5L-β) contain acetyl-[LRLRG]_2_-CO_2_H peptide attached to an internal abasic sugar residue via anomeric C1′ carbon either in α- or in β-configuration.

Herein we designed and synthesized a series of metal-free bulge-loop inducing peptidyl-oligonucleotide conjugates (BCs) against the 3′ acceptor stem and TΨC arm of tRNA^Phe^ (see Figure 1 and Supplementary Figures S1 and 2). The presence of common secondary structural elements and its characteristic folding makes tRNA^Phe^ a desirable target for scientific discovery. Through central incorporation of a modified adenosine or abasic nucleotide, changes in loop size, peptide structure and its orientation, a library of 14 novel conjugates were designed, synthesized and fully characterized here. We report their hybridization properties and catalytic activity, alongside comprehensive analysis of their interactions with target RNA using molecular modeling stimulations. This work illuminates the importance of strict structural and dynamic considerations for molecular design of peptidyl-oligonucleotide conjugates attacking bulge-loop regions of RNA sequences. The structural insights gained here inform how duplex-constrained cleavage occurs, so that a single molecule catalyst can be designed to destroy manifold copies of target RNA in a specific sequence-controlled manner.

## MATERIALS AND METHODS

### Chemical, reagents, equipment and facilities

Oligonucleotides containing an aminohexyl-modified adenosine residue were acquired from ATDBio Ltd (Southampton, UK), and those incorporating an internal abasic nucleotide with aminohexyl linker attached at the C1’ position in α- or β- configuration were purchased from the Institute of Chemical Biology and Fundamental Medicine (Novosibirsk, Russian Federation). α- and β- aminohexyl abasic sugar phosphoramidite monomers were purchased from Link Technologies Ltd (Scotland, UK). Acetyl-[LRLRG]_2_-CO_2_H was purchased from Biomatik (Cambridge, Ontario, Canada). Reagents and materials were purchased from Sigma-Aldrich (UK), unless otherwise indicated. Water was purified in house via a Milli-Q purification system (Millipore, USA).

HPLC purifications were carried out using an Agilent 1100 HPLC system (Agilent Technologies, Santa Clara, CA, USA) equipped with a diode array detector and Rheodyne (3725i) manual injector. Peptides and peptidyl-oligonucleotide conjugates were purified by reverse phase chromatography using a semipreparative Phenomenex Luna C-18 column (5 μm, 4.6 × 10 × 250 mm, 100 A, Phenomenex; CA, USA), with a flow rate of 2 ml min^−1^. All peptidyl-oligonucleotide conjugates were additionally purified by gel filtration chromatography using Illustra^™^ NAP disposable columns, prepacked with Sephadex G-25 (DNA grade) and purchased from GE Healthcare Life Sciences, to remove possible traces of counter cations. UV-visible spectroscopy measurements were carried out using a Cary-4000 UV-Visible spectrophotometer from Varian (Australia) connected to a Cary Peltier temperature controller, operating under Varian Cary WinUV software. Oligonucleotide concentrations were calculated by measuring UV absorbance at 260 nm and using the millimolar extinction coefficient ϵ_260_ of the corresponding oligonucleotide at 260 nm (mM^−1^·cm^−1^) (see Supplementary Table S1). ESI mass spectral data was collected on a Thermo Scientific LTQ Orbitrap XL (MA, USA) at the EPSRC UK National Mass Spectrometry Facility at Swansea University (Swansea, UK). MALDI (Matrix-Assisted Laser Desorption Ionisation) mass spectra was collected on a Bruker Dal-tonics Ultraflex TOF/TOF mass spectrometer (MA, USA) at the Manchester Interdisciplinary Biocentre, University of Manchester as described earlier (34). ^1^H NMR spectra were recorded using Bruker Avance II+ spectrometers operating at proton frequencies of 400 MHz using BBI ^1^H/D-BB Z-GRD Z8202/0347 probe using previously described methods (34,35). The acquired data was processed using Bruker software Topspin 4.0.3.

The analytical data presenting the full characterization of the generated peptides and peptidyl-oligonucleotide conjugates are given in the Supplementary Information (see Supplementary Figures S3-S12).

### Peptide synthesis, purification and characterization

Acetyl-[LR]_4_G-CO_2_H was synthesized by manual solid-phase methodology on Fmoc-Gly-Wang resin (250 mg, 0.2 mmol) using the Fmoc/tBu strategy. Fmoc deprotections were achieved with 20% piperidine in N,N-dimethylformamide (DMF) solution for 30 min at room temperature (RT). Before each coupling step, resin was washed with DMF (2 × 10 ml), dichloromethane (DCM) (2 × 10 ml) and DMF (2 × 10 ml). Amino acid couplings were achieved by pre-activating either Fmoc-Leu-OH (212 mg, 0.6 mmol); Fmoc-Gly-OH (178 mg, 0.54 mmol) or Fmoc-Arg(Pbf)-OH (389 mg, 0.6 mmol) in DMF (10 ml) with N,N,N′,N′-tetramethyl-O-(1H-benzotriazol-1-yl) uronium hexafluorophosphate (HBTU) (220 mg, 0.58 mmol) and N,N-di-isopropylethylamine (DIPEA) (122 μl, 0.7 mmol). Following addition to the resins, the reactions proceeded at RT for 30 min under mechanical shaking. Following completion of the peptide sequence, the N-terminus was acetylated by shaking with acetic anhydride (10 mmol) and DIPEA (10 mmol) in DMF (10 ml) for 30 min, followed by washing of the resin with DMF (2 × 10 ml), DCM (2 × 10 ml) and DMF (2 × 10 ml). Successful Fmoc deprotection and amino acid coupling was visualized by carrying out a Kaiser test. After completing the acetylation procedure, the peptide was cleaved from the resin by shaking with the mixture trifluoroacetic acid (TFA): triisopropyl silane (TIPS):H_2_O (95:2.5:2.5) (15 ml) at RT for 3 h. The volume of the resulting solution was reduced by 66%, pipetted into cold (−20°C) methyl tert-butyl ether (TBME) and stored at −20°C overnight. The precipitate was collected by centrifugation (4000 rpm, 5 min, 4°C) and the resulting pellets were washed with cold TBME (2 × 10 ml) and isolated by centrifugation. Once air dried, the pellets were solubilized in acetonitrile/water/TFA (40/60/0.1) and freeze dried.

Crude lyophilized peptide was solubilized in 30% acetic acid and purified using reverse-phase HPLC (RP-HPLC). The flow rate was maintained at 1.5 ml/min using 0.1% TFA/water as eluent A and 0.1% TFA/acetonitrile (AcCN) as eluent B. The absorbance was monitored at 220 nm, while the following gradient was applied: 5% B for 1 min, 5% B to 40% B in 40 min.

#### Acetyl-[LR]_4_G-CO_2_H

Fractions at 33 min were collected, combined and lyophilized to yield the TFA salt of peptide as a fluffy white material (240 mg, 73%). ESI-MS: m/z = 597.9 for [M+H+H]^2+^ (MW = 1193.78 calc. for [C_52_H_99_N_21_O_11_]) (Supplementary Figure S5). ^1^H NMR (Supplementary Figure S6) (D_2_O with TSP (0.1 μM), 400 MHz): δ 0.85- 0.96 (m, 24H, Leu-H^δ^), 1.50–1.93 (m, 28H, 8 × Arg-H^β^, 8 × Arg-H^γ^, 8 × Leu-H^β^, 4 × Leu-H^γ^), 2.03 (s, 3H, Ac-CH_3_), 3.20 (m, 8H, Arg-H^δ^), 3.85 (d, 2H, 2 × Gly-H), 4.15–4.38 (m, 8H, 8 × Leu/Arg-H^α^).

#### Acetyl-[LRLRG]_2_-CO_2_H

Fractions at 33 min were collected, combined and lyophilized to yield the TFA salt of the peptide as a fluffy white material. MALDI-TOF-MS: m/z = 1252 for [M+H]^+^, (MW = 1251 calc. for [C_54_H_102_N_22_O_12_]) (Supplementary Figure S7). ^1^H NMR (Supplementary Figure S8) (D_2_O with TSP (0.1 μM), 400 MHz): δ 0.91–0.97 (m, 24H, Leu-H^δ^), 1.55- 1.99 (m, 28H, 8 × Arg-H^β^, 8 × Arg-H^γ^, 8 × Leu-H^β^, 4 × Leu-H^γ^), 2.06 (s, 3H, Ac-CH_3_), 3.24 (m, 8H, Arg-H^δ^), 3.88–4.41 (m, 12H, 4 × Gly-H, 8 × Leu/Arg-H^α^).

### Conjugate synthesis

#### Type 1 peptidyl-oligonucleotide conjugates (BC2-BC5)

Oligonucleotides containing an aminohexyl-modified adenosine residue (50 nmol) in H_2_O (100 μl) were converted into dimethyl sulfoxide (DMSO) soluble salts through multiple (10 μl) additions of a 4% (w/v) cetyltrimethylammonium bromide (CTAB) solution. After each subsequent addition, the solution was centrifuged (13400 rpm, 4 min). When oligonucleotide precipitation had completed, the pellet was harvested by centrifugation and washed with H_2_O (2 × 500 μl) and freeze dried overnight. The pellet was solubilized in anhydrous DMSO (50 μl). Peptide acetyl-[LR]_4_G-CO_2_H (2.3 μmol) and 4-dimethylaminopyridine (DMAP, 3.5 μmol) were dissolved in a minimum volume of anhydrous DMSO (≈20 μl) before N, N'-dicyclohexylcarbodiimide (DCC, 3.5 μmol) was added and vortexed. The resulting peptide solution was added directly to oligonucleotide solutions and left at 40°C for 4 hours. Lithium perchlorate (LiClO_4_, 4% w/v) in acetone (1.8 ml) was added and the reaction mixture left at −80°C overnight. After centrifugation (13 400 rpm, 4 min), the supernatant was discarded and the pellet left to air dry. The dry pellet was vortexed vigorously with 3M LiClO_4_ (120 μl) and centrifuged (13 400 rpm, 4 min). The supernatant was carefully transferred into 4% LiClO_4_ in acetone (w/v) (1.8 ml) and kept at −80°C for 3 hours. Following centrifugation (13 400 rpm, 4 min), the supernatant was discarded and the pellet left to air dry before RP-HPLC purification.

#### Type 2 conjugates (BC-α and BC-βs)

The water-soluble lithium salt of the oligonucleotide was converted to its CTAB (50 nmol) salt (to solubilize in DMSO) using the same protocol as described for Type 1 conjugates. Following lyophilization, the dry pellet was solubilized in 30 μl of anhydrous DMSO. In an attempt to achieve complete dissolution in the minimum of DMSO, the oligonucleotide was vigorously vortexed and heated up to 60°C. Subsequently, dried acetyl-[LRLRG]_2_-CO_2_H (50-fold excess over oligonucleotide, 2.5 μmol), DMAP and DCC (1.5-fold excess over peptide each, 3.75 μmol) were dissolved in the minimum volume of anhydrous DMSO (≈40 μl) and vortexed. The resulting solution was then added directly to oligonucleotide solution and incubated at 60°C for 12 h. Subsequently, LiClO_4_ (1.8 ml of 4% w/v) in acetone was added directly to the reaction vessel and left at −80°C for 36 h. Following centrifugation (13 400 rpm, 5 min), the supernatant was decanted when the precipitate was left to air dry. The dry pellet was dissolved in water (2 × 1 ml) and prepared for size-exclusion chromatography before purification through RP-HPLC. Where different amounts of the oligonucleotide were used, the amounts of peptide, DMAP and DCC were scaled accordingly to maintain the same molar ratios.

### Conjugate purification

#### Purification of Type 1 conjugates

Crude conjugates were dissolved in 0.05M LiClO_4_ and purified by RP-HPLC. The flow rate was maintained at 2.0 ml/min using 0.05M LiClO_4_ as eluent A and 0.05M LiClO_4_ in AcCN as eluent B. The absorbance was monitored at 260 nm and the following gradient was applied: 100% A for 5 min, 0% B to 40% B in 27 min (see Supplementary Figure S3).

#### Purification of Type 2 conjugates

Crude conjugates were dissolved in water and purified using RP-HPLC. The flow rate was maintained at 2.0 ml/min using 0.05M LiClO_4_ as eluent A and 0.05M LiClO_4_ in AcCN as eluent B. The absorbance was monitored at 260 nm and the following gradient was applied: 0% B for 3 min, 0–100% B in 30 min (see Supplementary Figure S4).

### Conjugate characterization

#### BC2

Fractions at 27 min were collected, combined and lyophilized. The excess salt was removed by dissolving the material in H_2_O (100 μl) and precipitating overnight in LiClO_4_ in acetone (1.8 ml of 4% w/v). The product was collected by centrifugation (13 400 rpm, 4 min), air dried and then freeze dried to give a white powder (24 nmol, 48%). MALDI-MS: *m/z* = 4088 [M+H+Na]^2+^, 8184 for [M+K]^+^ (MW = 8144 calc. for [C_276_H_382_N_111_O_140_P_21_]) (Supplementary Figure S9). ^1^H NMR (Supplementary Figure S10) (D_2_O with TSP (0.01 mM), 400 MHz): δ 0.70–0.99 (m, 24H, Leu- H^δ^), 0.85–3.25 (m, 105H, 22 × H2’ and 22 × H2’’ sugar ring protons, 6 × CH_3_ of 6 × dT, 8 × Arg-H^β^, 8 × Arg-H^γ^, 8 × Leu-H^β^, 4 × Leu-H^γ^, 6 × CH_2_ (aminohexyl linker), acetyl-CH_3_), 3.21 (m, 8H, 8 × Arg-H^δ^), 3.51–4.23 (m, 76H, 22 × H4’/H5’H5’ sugar ring protons, 2 × Gly-H, 8 × Leu/Arg-H^α^), 4.52–4.94 (m, 22H, 22 × H3’ sugar ring protons), 5.40–6.33 (m, 24H, 22 × H1’ sugar ring protons, 2 × H5 of dC), 7.25–8.31 (m, 27H, 27 × Ar-H from dG(H8 × 8), dA(H8 × 5), dA(H2 × 6), dC(H6 × 2) and dT(H6 × 6). H3’ sugar ring protons (4.3–5.2 ppm) were not analyzed due to suppression of residual water signal at 4.78 ppm.

#### BC3

Fractions at 26 min were collected, combined and lyophilized. The excess salt was removed by dissolving the material in H_2_O (100 μl) and precipitating in 4% LiClO_4_ in acetone (w/v) (1.8 ml) overnight. The product was collected by centrifugation (13 400 rpm, 4 min), air-dried and then freeze-dried to give a white powder (19 nmol, 38%). MALDI-MS: *m/z* = 3919 [M+H+Na]^2+^,7816 for [M+K]^+^ (MW = 7815 calc. for [C_266_H_370_N_106_O_134_P_20_]) (Supplementary Figure S9). ^1^H NMR (Supplementary Figure S10) (D_2_O with TSP (0.01 mM), 400 MHz): δ 0.70–0.99 (m, 24H, Leu- H^δ^), 0.85–3.25 (m, 103H, 21 × H2’ and 21 × H2’’ sugar ring protons, 6 × CH_3_ of 6 × dT, 8 × Arg-H^β^, 8 × Arg-H^γ^, 8 × Leu-H^β^, 4 × Leu-H^γ^, 6 × CH_2_ (aminohexyl linker), acetyl-CH_3_), 3.21 (m, 8H, 8 × Arg-H^δ^), 3.51–4.23 (m, 73H, 21 × H4’/H5’H5’ sugar ring protons, 2 × Gly-H, 8 × Leu/Arg-H^α^), 5.40–6.33 (m, 23H, 21 × H1’ sugar ring protons, 2 × H5 of dC), 7.25–8.31 (m, 26H, 26 × Ar-H from dG(H8 × 7), dA(H8 × 5), dA(H2 × 6), dC(H6 × 2) and dT(H6 × 6)). H3’ sugar ring protons (4.3–5.2 ppm) were not analyzed due to suppression of residual water signal at 4.78 ppm.

#### BC4

Fractions at 26.5 min were collected, combined and lyophilized. The excess salt was removed by dissolving the material in H_2_O (100 μl) and precipitating overnight in LiClO_4_ (1.8 ml of 4% w/v in acetone). The product was collected by centrifugation (13 400 rpm, 4 min), air dried and then freeze dried to give a white powder (22 nmol, 44%). MALDI-MS: *m/z* = 3790 [M+2H+2ACN]^2+^, 7541 for [M+Na+Na-H]^+^ (MW = 7497 calc. for [C_256_H_357_N_104_O_127_P_19_]) (Supplementary Figure S9). ^1^H NMR (Supplementary Figure S10) (D_2_O with TSP (0.01 mM), 400 MHz): δ 0.70–0.99 (m, 24H, Leu-H^δ^), 0.85–3.25 (m, 98H, 20 × H2’ and 20 × H2’’ sugar ring protons, 5 × CH_3_ of 5 × dT, 8 × Arg-H^β^, 8 × Arg-H^γ^, 8 × Leu-H^β^, 4 × Leu-H^γ^, 6 × CH_2_ (aminohexyl linker), acetyl-CH_3_), 3.21 (m, 8H, 8 × Arg-H^δ^), 3.51–4.23 (m, 70H, 20 × H4’/H5’H5’ sugar ring protons, 2 × Gly-H, 8 × Leu/Arg-H^α^), 5.40–6.33 (m, 22H, 20 × H1’ sugar ring protons, 2 × H5 of dC), 7.25–8.31 (m, 25H, 25 × Ar-H from dG(H8 × 7), dA(H8 × 5), dA(H2 × 6), dC(H6 × 2) and dT(H6 × 5)). H3’ sugar ring protons (4.3–5.2 ppm) were not analyzed due to suppression of residual water signal at 4.78 ppm.

#### BC5

Fractions at 27 min were collected, combined and lyophilized. The excess salt was removed by dissolving the material in H_2_O (100 μl) and precipitating overnight in LiClO_4_ (1.8 ml of 4% w/v in acetone). The product was collected by centrifugation (13 400 rpm, 4 min), air dried and then freeze dried to give a white powder (19 nmol, 38%). MALDI-MS: *m/z* = 3603 [M+H+K]^2+^,7201 for [M+CH3OH+H]^+^ (MW = 7168 calc. for [C_247_H_347_N_99_O_121_P_18_]) (Supplementary Figure S9). ^1^H NMR (Supplementary Figure S10) (D_2_O with TSP (0.01 mM), 400 MHz): δ 0.70–0.99 (m, 24H, Leu-H^δ^), 0.85–3.25 (m, 96H, 19 × H2’ and 19 × H2’’ sugar ring protons, 5 × CH_3_ of 5 × dT, 8 × Arg-H^β^, 8 × Arg-H^γ^, 8 × Leu-H^β^, 4 × Leu-H^γ^, 6 × CH_2_ (aminohexyl linker), acetyl-CH_3_), 3.21 (m, 8H, 8 × Arg-H^δ^), 3.51–4.23 (m, 67H, 20 × H4’/H5’H5’ sugar ring protons, 2 × Gly-H, 8 × Leu/Arg-H^α^), 5.40–6.33 (m, 21H, 19 × H1’ sugar ring protons, 2 × H5 of dC), 7.25–8.31 (m, 24H, 24 × Ar-H from dG(H8 × 6), dA(H8 × 5), dA(H2 × 6), dC(H6 × 2) and dT(H6 × 5)). H3’ sugar ring protons (4.3–5.2 ppm) were not analyzed due to suppression of residual water signal at 4.78 ppm.

#### BC2-α

MALDI-ToF: *m/z* = 9636 [M+2Na] ^+^ (MW = 9590 calc. For [C_320_H_439_N_127_O_170_P_26_) (Supplementary Figure S11). ^1^H NMR (Supplementary Figure S12) (D_2_O with TSP (0.01 mM), 400 MHz): δ 0.77–0.87 (m, 24H, Leu- H^δ^), 1.05–2.72 (m, 115H, 27 × H2’ and 27 × H2’’ sugar ring protons, 6 × CH_3_ of 6 × dT, 8 × Arg-H^β^, 8 × Arg-H^γ^, 8 × Leu-H^β^, 4 × Leu-H^γ^, 6 × CH_2_ (aminohexyl linker), acetyl-CH_3_), 3.16 (m, 8H, 8 × Arg-H^δ^), 3.22–4.66 (m, 93H, 81 × H4’/H5’H5’ sugar ring protons, 2 × Gly-CH_2_, 8 × Leu/Arg-H^α^), 4.85–6.27 (m, 31H, 27 × H1’ sugar ring protons, 4 × H5 of dC), 7.25–8.42 (m, 33H, 33 × Ar-H from dG(H8 × 9), dA(H8 × 7), dA(H2 × 7), dC(H6 × 4) and dT(H6 × 6)). H3’ sugar ring protons (4.3–5.2 ppm) were not analyzed due to suppression of residual water signal at 4.76 ppm. Not all H4’/H5’H5’ sugar ring protons were fully resolved, and suppression of residual water prevented signal detection.

#### BC3-α

MALDI-ToF: *m/z* = 9305 [M+Na+Li] ^+^ (MW = 9275 calc. For [C_311_H_429_N_122_O_164_P_25_]) (Supplementary Figure S11). ^1^H NMR (Supplementary Figure S12) (D_2_O with TSP (0.01 mM), 400 MHz): δ 0.81–0.90 (m, 24H, Leu- H^δ^), 1.12–3.09 (m, 113H, 26 × H2’ and 26 × H2’’ sugar ring protons, 6 × CH_3_ of 6 × dT, 8 × Arg-H^β^, 8 × Arg-H^γ^, 8 × Leu-H^β^, 4 × Leu-H^γ^, 6 × CH_2_ (aminohexyl linker), Acetyl-CH_3_), 3.21 (m, 8H, 8 × Arg-H^δ^), 3.29–4.74 (m, 90H, 78 × H4’/H5’H5’ sugar ring protons, 2 × Gly-CH_2_, 8 × Leu/Arg-H^α^), 4.92–6.24 (m, 30H, 26 × H1’ sugar ring protons, 4 × H5 of dC), 7.26–8.44 (m, 32H, 32 × Ar-H from dG(H8 × 8), dA(H8 × 7), dA(H2 × 7), dC(H6 × 4) and dT(H6 × 6)). H3’ sugar ring protons (4.3–5.2 ppm) were not analyzed due to suppression of residual water signal at 4.76 ppm.

#### BC4-α

MALDI-ToF: *m/z* = 9003 [M+2Na] ^+^ (MW = 8957 calc. For [C_300_H_414_N_120_O_157_P_24_]) (Supplementary Figure S11). ^1^H NMR (Supplementary Figure S12) (D_2_O with TSP (0.01 mM), 400 MHz): δ 0.82–0.89 (m, 24H, Leu- H^δ^), 1.05–2.83 (m, 108H, 25 × H2’ and 25 × H2’’ sugar ring protons, 5 × CH_3_ of 5 × dT, 8 × Arg-H^β^, 8 × Arg-H^γ^, 8 × Leu-H^β^, 4 × Leu-H^γ^, 6 × CH_2_ (aminohexyl linker), acetyl-CH_3_), 3.17 (m, 8H, 8 × Arg-H^δ^), 3.24–4.69 (m, 87H, 75 × H4’/H5’H5’ sugar ring protons, 2 × Gly-CH_2_, 8 × Leu/Arg-H^α^), 4.91–6.29 (m, 29H, 25 × H1’ sugar ring protons, 4 × H5 of dC), 7.26–8.45 (m, 31H, 31 × Ar-H from dG(H8 × 8), dA(H8 × 7), dA(H2 × 7), dC(H6 × 4) and dT(H6 × 5)). H3’ sugar ring protons (4.3–5.2 ppm) were not analyzed due to suppression of residual water signal at 4.32 ppm.

#### BC5-α

MALDI-ToF: *m/z* = 8666 [M+K] ^+^ (MW = 8627 calc. For [C_290_H_402_N_115_O_151_P_23_]) (Supplementary Figure S11). ^1^H NMR (Supplementary Figure S12) (D_2_O with TSP (0.01 mM), 400 MHz): δ 0.81–0.90 (m, 24H, Leu- H^δ^), 1.20–2.40 (m, 106H, 24 × H2’ and 24 × H2’’ sugar ring protons, 5 × CH_3_ of 5 × dT, 8 × Arg-H^β^, 8 × Arg-H^γ^, 8 × Leu-H^β^, 4 × Leu-H^γ^, 6 × CH_2_ (aminohexyl linker), acetyl-CH_3_), 3.18 (m, 8H, 8 × Arg-H^δ^), 3.52–4.41 (m,84H, 72 × H4’/H5’H5’ sugar ring protons, 2 × Gly- CH_2_, 8 × Leu/Arg-H^α^), 5.50–6.26 (m, 28H, 24 × H1’ sugar ring protons, 4 × H5 of dC), 7.25–8.45 (m, 30H, 30 × Ar-H from dG(H8 × 7), dA(H8 × 7), dA(H2 × 7), dC(H6 × 4) and dT(H6 × 5)). Unfortunately, low signal-to-noise and distortion in nuclear magnetic resonance (NMR) signals prevented full assignment of all peaks and resulted in presence of extra signals. H3’ sugar ring protons (4.3–5.2 ppm) were not analyzed due to suppression of residual water signal at 4.30 ppm.

#### BC5L-α

MALDI-MS: *m/z* = 9605 [M+2Na] ^+^ (MW = 9559 calc. For [C_319_H_438_N_128_O_168_P_26_]) (Supplementary Figure S11). ^1^H NMR (Supplementary Figure S12) (D_2_O with TSP (0.01 mM), 400 MHz): δ 0.80–0.85 (m, 24H, Leu- H^δ^), 1.20–2.56 (m, 112H, 27 × H2’ and 27 × H2’’ sugar ring protons, 5 × CH_3_ of 5 × dT, 8 × Arg-H^β^, 8 × Arg-H^γ^, 8 × Leu-H^β^, 4 × Leu-H^γ^, 6 × CH_2_ (aminohexyl linker), acetyl-CH3), 3.17 (m, 8H, 8 × Arg-H^δ^), 3.51–4.40 (m,93H, 81 × H4’/H5’H5’ sugar ring protons, 2 × Gly- CH_2_, 8 × Leu/Arg-H^α^), 4.84–6.36 (m, 32H, 27 × H1’ sugar ring protons, 5 × H5 of dC), 7.24–8.45 (m, 34H, 34 × Ar-H from dG(H8 × 8), dA(H8 × 8), dA(H2 × 8), dC(H6 × 5) and dT(H6 × 5)). Unfortunately, low signal-to-noise and distortion in NMR signals prevented full assignment of all peaks and resulted in presence of extra signals. A slight shift in the position of sugar ring protons was witnessed. H3’ sugar ring protons (4.3–5.2 ppm) were not analyzed due to suppression of residual water signal at 4.30 ppm.

#### BC3-β

MALDI-MS: *m/z* = 9304 [M+Na+Li] ^+^ (MW = 9275 calc. For [C_311_H_429_N_122_O_164_P_25_]) (Supplementary Figure S11). ^1^H NMR (Supplementary Figure S12) (D_2_O with TSP (0.01 mM), 400 MHz): δ 0.81–0.90 (m, 24H, Leu- H^δ^), 1.12–2.55 (m, 113H, 26 × H2’ and 26 × H2’’ sugar ring protons, 6 × CH_3_ of 6 × dT, 8 × Arg-H^β^, 8 × Arg-H^γ^, 8 × Leu-H^β^, 4 × Leu-H^γ^, 6 × CH_2_ (aminohexyl linker), acetyl-CH_3_), 3.17 (m, 8H, 8 × Arg-H^δ^), 3.54–4.39 (m, 90H, 78 × H4’/H5’H5’ sugar ring protons, 2 × Gly- CH_2_, 8 × Leu/Arg-H^α^), 4.92–6.24 (m, 30H, 26 × H1’ sugar ring protons, 4 × H5 of dC), 7.25–8.44 (m, 32H, 32 × Ar-H from dG(H8 × 8), dA(H8 × 7), dA(H2 × 7), dC(H6 × 4) and dT(H6 × 6)). H3’ sugar ring protons (4.3–5.2 ppm) were not analyzed due to suppression of residual water signal at 4.76 ppm.

#### BC5L-β

MALDI-MS: *m/z* = 9606 [M+2Na] ^+^ (MW = 9559 calc. For [C_319_H_438_N_128_O_168_P_26_]) (Supplementary Figure S11). ^1^H NMR (Supplementary Figure S12) (D_2_O with TSP (0.01 mM), 400 MHz): δ 0.81–0.88 (m, 24H, Leu- H^δ^), 1.20–2.54 (m, 112H, 27 × H2’ and 27 × H2’’ sugar ring protons, 5 × CH_3_ of 5 × dT, 8 × Arg-H^β^, 8 × Arg-H^γ^, 8 × Leu-H^β^, 4 × Leu-H^γ^, 6 × CH_2_ (aminohexyl linker), acetyl-CH_3_), 3.17 (m, 8H, 8 × Arg-H^δ^), 3.38–4.20 (m,93H, 81 × H4’/H5’H5’ sugar ring protons, 2 × Gly- CH_2_, 8 × Leu/Arg-H^α^), 4.37–6.26 (m, 32H, 27 × H1’ sugar ring protons, 5 × H5 of dC), 7.25–8.44 (m, 34H, 34 × Ar-H from dG(H8 × 8), dA(H8 × 8), dA(H2 × 8), dC(H6 × 5) and dT(H6 × 5)). H3’ sugar ring protons (4.3–5.2 ppm) were not analyzed due to suppression of residual water signal at 4.10 ppm.

### Preparation of linearized plasmids and *in vitro* RNA transcripts

Preparation of linearized plasmid p67YF0 as well as *in vitro* transcript of yeast tRNA^Phe^ were as reported earlier (15). 3′-end labeling of RNA transcript with fluorescein isothiocyanate (FITC) and 5′-labeling of RNA fragments with [γ-^32^P]ATP were carried out as reported (15,36).

### Hybridization of 3′-FITC tRNA^Phe^ with BCs

The hybridization assays between 3′-FITC-tRNA^Phe^ at concentration 1 μM and one of the BCs at concentrations varying from 0.25 to 10 μM, as well as subsequent quantitative data analysis were carried out as described earlier (15).

### Cleavage of 3′-FITC tRNA^Phe^ with BCs

Cleavage experiments were carried out for 3′-FITC labeled tRNA^Phe^ at 1 μM (single turnover conditions) and one of the BCs at concentration 20 μM in 50 mM Tris–HCl pH 7.0, 0.2 M KCl, 1 mM ethylenediaminetetraacetic acid (EDTA) according to the previously reported protocols (15).

### Ribonuclease H cleavage assay

The reaction mixture (5 μl) contained 3′-FITC-tRNA^Phe^ (1 μM), one of the bulge-inducing conjugates (at 40 μM concentration), 50 mM Tris–HCl pH 7.0, 0.2 M KCl and 1 mM EDTA. In parallel, the control experiment was carried out under identical conditions, but with 3′-FITC-tRNA^Phe^ alone. Considerable excess of the conjugate (BC2, BC3, BC4 or BC5) was used over 3′-FITC-tRNA^Phe^ (40:1) to ensure efficient hybridization prior to treatment with ribonuclease H. The mixtures were incubated at 37°C for 30 min. RNase H (1U) was added to BC:RNA complexes and incubated at 37°C for 15 min. The reactions were quenched by RNA precipitation with 2% (w/v) lithium perchlorate in acetone (75 μl). RNA pellet was collected by centrifugation and dissolved in loading buffer (8 M urea, 0.025% bromophenol blue, 0.025% xylene cyanol). RNA cleavage products were resolved in 12% polyacrylamide/8 M urea gel electrophoresis using Tris/Borate/EDTA (TBE) as running buffer. To identify cleavage sites, an imidazole ladder and an RNase T1-ladder produced by partial tRNA^Phe^ cleavage with 2 M imidazole buffer (pH 7.0) and RNase T1, respectively, were run in parallel. The gel was analyzed using ChemiDoc-MP (Bio-Rad).

### RNase A probing of tRNA^Phe^/bulge-inducing conjugates complex

Prior to cleavage with RNase A under physiological conditions, the complex of 3′-FITC-tRNA^Phe^ (1 μM) with one of the bulge-inducing conjugates (20 μM) was pre-formed in 10 μl of 50 mM Tris–HCl, pH 7.0, 0.2 M KCl, 1 mM EDTA and 100 μg/ml total tRNA from *Escherichia coli* as carrier and maintained at 37°C for 20 min. Subsequently, RNase A was added to the reaction mixture to achieve 1 nM final concentration, and the cleavage reaction was carried out for 10 min at 37°C. The reactions were quenched by RNA precipitation with 100 μl of 2% lithium perchlorate in acetone. RNA was collected by centrifugation and dissolved in loading buffer (8 M urea, 0.025% bromophenol blue, 0.025% xylene cyanol). RNA cleavage products were resolved in 12% polyacrylamide/8 M urea gel using TBE (15) as running buffer. To identify cleavage sites, an imidazole ladder and an RNase T1-ladder produced by partial tRNA^Phe^ cleavage with 2 M imidazole buffer (pH 7.0) and RNase T1, respectively, were run in parallel. The gel was analyzed using PharosFX Plus System (Bio-Rad).

### Molecular modeling

Computational studies were performed with AMBER (37) (AMBER 16 and AMBERTOOLS 16) gaussian 09 (38)^,^ GaussView6 (39) and Chimera (40) molecular modeling packages.

#### Generation of the initial structures

The initial 3D structures of the complexes between the tRNA fragment and peptidyl-oligonucleotide conjugates were generated via assembly of distinct components. The *RNA–DNA* hybrid double helices were modeled in the Type-A form using the *Make-NA* online server (http://structure.usc.edu/make-na/server.htm). The peptide was created initially in an extended conformation, and the connection to the DNA structure was achieved using special nucleotides: ADX (Type 1), the abasic residues AXA ‘alpha’ or AXB ‘beta’ (Type 2) and the linkers N6N (Type 1) and N6O (Type 2). By combining these components with the appropriate RNA and DNA chains, all the systems were built with the required specifications of stereochemistry and geometry.

The linkers and special residues were created following the RESP methodology in AMBER (41). The geometries were first optimized, and the electrostatic potential was calculated using Gaussian09 Hartree–Fock method and the 6–31G* basis set. The geometries and ESP charges were processed with antechamber and RESP Amber modules to obtain the restrained ESP charges, in order to safely remove the capping groups from the units. Finally, the oligonucleotide was built, and the components of the complex assembled via xLEaP. Parameters were assigned to the DNA, RNA, special residues ADX, AXA, AXB and peptide from the ff14SB (42) (explicit water model) and ff14SBonlysc (implicit solvent model) while the GAFF force field (43) was used for the linkers N6N and N6O.

#### Molecular dynamics simulations

The molecular modeling simulations included series of Simulated Annealing (SA) steps using a molecular dynamics (MD) engine to optimally explore the conformational space and generate an ensemble of conformations.

##### Structure preparation

The lack of available X-ray or NMR experimental data on such structures makes the election of input coordinates a challenging task. The initial configurations were designed in house after discussions with the experimental team. A hybrid double helix RNA–DNA (44) was used in order to describe the complex state during the cleavage.

##### Simulated annealing protocol

The next stage consisted of a conformational search by rounds of 100 restrained SA steps in implicit solvent. The mechanism of action of the conjugates involves the peptide approaching the DNA to catalyze the bond break. In order to model this process, an ensemble of conformations of the peptide was generated by maintaining the *DNA–RNA* complex fixed with soft harmonic positional restrains and by running a sequence of SA steps to allow the peptide exploring the surface available at reach distance.

Each SA step consisted of a chain of four MD simulations with a total time of 11 ns: (i) a heating ramp over 0.5 ns, when the temperature was brought from 300 K to 800 K, (ii) an equilibration over 5.0 ns at 800K, (iii) a cooling ramp over 0.5 ns, when the temperature was lowered from 800 K to 300 K and (iv) an equilibration over 5 ns at 300 K. In each SA step the Generalized Born (GB) implicit solvent model (45–47) was used (igb = 1). Salt concentration was set to 0.1 M with the Debye–Hückel salt concentration model. The temperature coupling with the external bath was modeled using the Andersen thermostat (48,49). The SHAKE algorithm was applied to constrain all bonds containing hydrogen atoms. The equations of motion were propagated with a timestep of 1 fs. The non-bonded interactions were truncated using a cutoff value of 9999.0 Å.

Soft harmonic positional restrains with a force constant of 0.01 kcal⋅mol^−1^⋅Å^−1^ were applied to a group of nucleotides to keep the complex in place. The low value of the force constant helps the system to smoothly adapt to the heating-cooling SA process. The nucleotides in the vicinity of the special residue bridging the DNA with the peptide and the ones in the tail of the DNA were not retrained to ensure a proper conformational exploration. Every complex was annealed for 100 times and the structures obtained was used in the next stage for analysis.

##### Cluster analysis

The geometrical analysis of the ensembles of structures was achieved using ptraj/cpptraj modules in AMBER 16, and the visualization and clustering was carried out with Chimera set of tools. In order to cluster the obtained *Conjugate-RNA* structures into conformationally related subfamilies, which are most different from each other in terms of RMSD values of heavy atoms, we used Chimera software. This approach allows (i) automatically, systematically and rapidly clustering structures into a set of conformationally related subfamilies generated during the SA/MD simulations, and (ii) selects a representative structure from each cluster, as previously described in detail (40,50). The probability of *‘in-line’* conformation implementation for every phosphate in the bulge-loop region of the *RNA-Conjugate* hybrid was calculated as the ratio between the number of structures with θ > 155° in MD trajectory to the overall number of all frames. The distance distribution between the phosphorus atom or O2′ atom in the bulge region and the nitrogen atoms in guanidinium group of all four arginine residues was calculated for the frames of the productive trajectories.

## RESULTS AND DISCUSSION

### Design and synthesis of BCs, BC-α and BC-β

Figure 1A and B illustrates the general concept of structural design, common for all bulge-loop inducing conjugates studied here against the 3′-acceptor stem and TΨC arm of tRNA^Phe^. Conjugates were synthesized with two different strategies for the attachment of the catalytic peptide: either via a centrally modified nucleotide (Type 1) or through an abasic sugar residue (Type 2) located in the middle of the RNA recognition motif (Figure 1C and D, respectively). Upon hybridization of such conjugates to the tRNA^Phe^, a bulge-loop of 2-nt, 3-nt, 4-nt or 5-nt size was expected to be formed around the ^61^C-A-C-A-G^65^ region of the tRNA^Phe^, while maintaining Watson–Crick hydrogen-bonding with the 3′-acceptor stem and TΨC loop/arm (see Figure 1 A and B). The induced bulge-loops regions were expected to be susceptible to cleavage by an RNA-cleaving peptide. Positioning the cleaving peptide in the middle of the oligonucleotide sequence paves the way to achieve catalytic turnover of RNA substrate. The number of base pairs stabilizing the *POC:RNA* heteroduplex is cut in half upon RNA cleavage, which favors dissociation of the POC from RNA after a cleavage event, and may promote competitive displacement of cleaved products by fresh target substrate.

Type 1 bulge-loop inducing conjugates (BC2–BC5) contained relatively short recognition motifs designed to hybridize mainly to the loop region of the TΨC arm, in addition to the 3′ acceptor stem of the tRNA^Phe^ (indicated by purple line in Figure 1A). By truncating the length of the oligonucleotide recognition motifs (from 23 to 22, 21, 20 or 19-mer) and by replacing cytidine residue at position 12 with aminohexyl modified adenosine A* (see Figure 1C and Supplementary Figure S1), non-complementary nucleobases of the tRNA strand (ranging from 0 to 2, 3, 4 or 5 residues, respectively) were induced at the position opposite to the peptide-modified adenosine A* within the hybridized complex. Amphiphilic peptide acetyl-[LR]_4_G-CO_2_H, which has previously demonstrated efficient cleavage of tRNA^Phe^*in vitro*,(15) was conjugated via its C-terminus to the aminohexyl linker of the A* nucleotide residue, as described in ‘Materials and Methods’ section.

Type 2 bulge-loop inducing conjugates were structurally different from Type 1 (Figure 1 D). First, the peptide was incorporated via an abasic sugar residue, through an aminohexyl linker attached to the anomeric C1’ carbon, either in α- or in β-configuration (see also Supplementary Figure S2). This approach provided us with the opportunity to generate two stereospecific series of Type 2 conjugates: BC-α (BC2-α, BC3-α, BC4-α, BC5-α and BC5L-α) and BC-β (BC2-β, BC3-β, BC4-β, BC5-β and BC5L-β). In order to explore the impact of enhanced hybridization on potency, Type 2 conjugates incorporated longer oligonucleotide recognition motifs than Type 1 conjugates, ranging from 23- to 26-mer (indicated by brown line in Figure 1A). Furthermore, BC5L-α and BC5L-β were elongated by three extra nucleotides to bind additionally the variable loop (indicated by arrows in Figure 1A) in order to compensate for the largest (5 nt) bulge-loop size. Finally, Type 2 conjugates carried an elongated catalytic peptide acetyl-[LRLRG]_2_-CO_2_H bearing an extra glycine residue in the middle of the chain. It has been shown earlier (16) that the elongated peptide can provide more efficient RNA cleavage, presumably due to higher degree of conformational flexibility. The conjugates within the same category differed from each other by the size of the bulge-loop they could induce, which is indicated in their nomenclature by the number of nucleotides involved.

These variations in structural design between and within Type 1 and Type 2 conjugates allowed us to investigate the impact on RNA cleavage of key factors, including: (i) the mode of peptide attachment and/or its configuration, (ii) the size of the induced single-stranded RNA bulge-loop region, (iii) the length and the structure of the catalytic peptide, (iv) the hybridization power of the oligonucleotide recognition motif and (v) the level of conformational flexibility. Molecular flexibility plays a vital role in catalysis, since a sufficient level of conformational freedom allows the substrate to adopt the reaction transition state (16). The use of a flexible aminohexyl linker between the oligonucleotide and peptide, and incorporation of the additional glycine in the peptide for Type 2 conjugates, allowed us to explore conformational possibilities that might favor catalysis (17).

Synthesis and full characterization of the peptides and conjugates are described in the ‘Materials and Methods’ section. HPLC analysis of Type 1 and Type 2 conjugates (Supplementary Figures S3 and 4) showed a consistent 4–5 min increase in retention time relative to unmodified oligonucleotides, thus confirming conjugation. The identity and purity of the peptides and synthesized conjugates were confirmed by ^1^H NMR spectroscopy, and mass spectrometry (see Supplementary Figures S5–12). ^1^H-NMR analysis (Supplementary Figures S10 and 12) of such large biomolecules containing 19–26 nt, 9 or 10 amino acids and aminohexyl linker is complicated by the large number of overlapping proton resonances, especially in the peptide and deoxyribose H2’/H2’/H4’/H5’/H5’ regions. Thus, unambiguous assignment of individual ^1^H NMR signals was not possible. However, comparison of the ^1^H NMR spectra of the conjugates and original oligonucleotides allowed us to confirm successful conjugation.

Characteristic peptide signals, corresponding to the Leu H^δ^ (0.8–1.0 ppm) and Arg-H^δ^ (3.1–3.3 ppm) protons were easily identifiable in the NMR spectra of the conjugates. Careful integration of these peptide protons, against the oligonucleotide aromatic region (7.26–8.44) and H1’ sugar ring protons, allowed us to confirm the 1:1 stoichiometric ratio of peptide attachment to the oligonucleotide (see Supplementary Figures S10 and 12). This was further verified by MALDI-ToF mass spectrometry of conjugates, which showed experimental masses in close agreement with the calculated values (see Supplementary Figures S9 and 11).

### Hybridization of bulge-inducing conjugates to tRNA^Phe^

**Figure 2.**
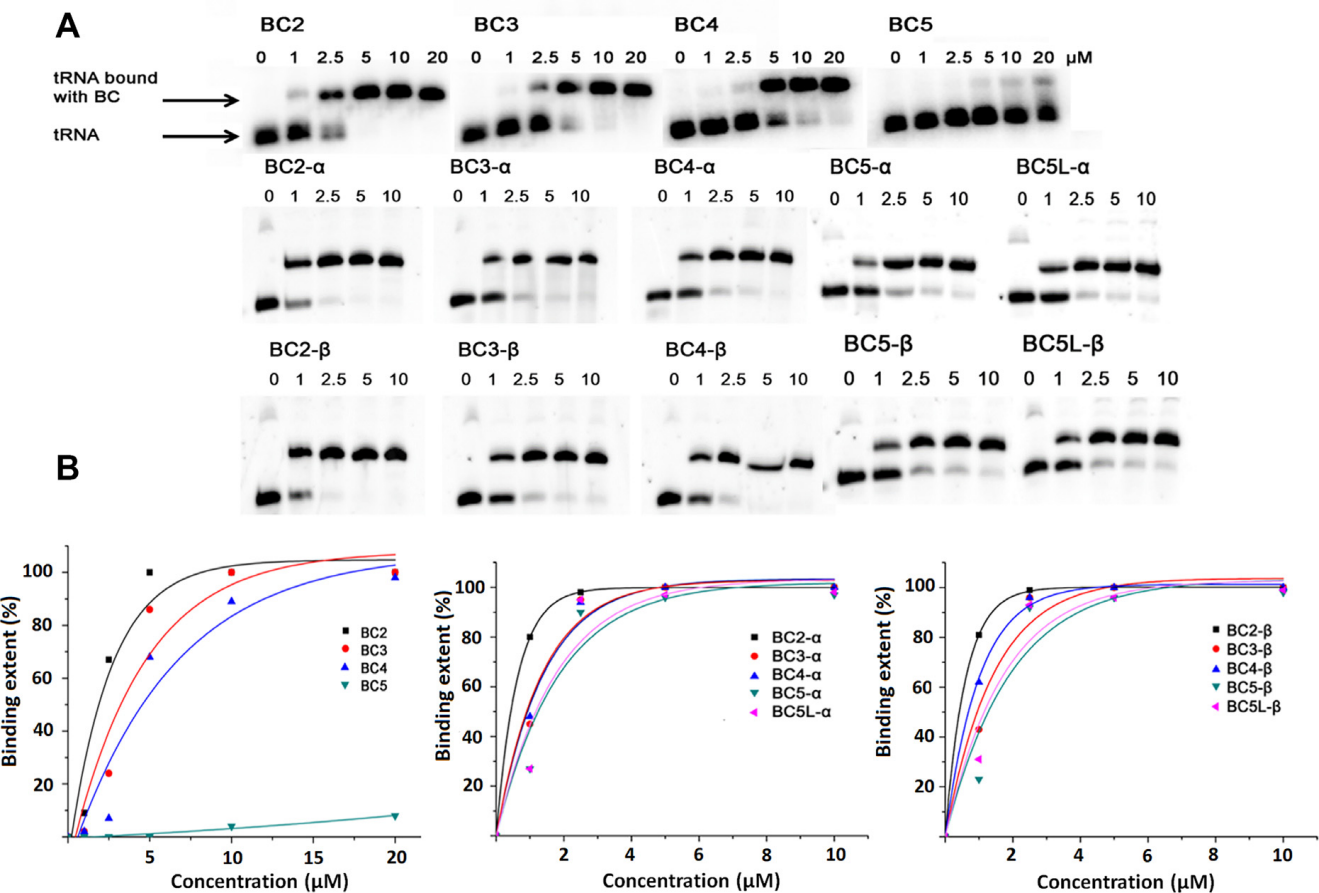
Gel-shift analysis of hybridization of bulge-loop inducing conjugates BCn, BCn-α and BCn-β with 3′-FITC-tRNA^Phe^. (**A**) Representative images of the PAGE showing binding of BCn, BCn-α and BCn-β at various concentrations (0–20 μM) with 3′-FITC-tRNA^Phe^ (1μM). (**B**) Secondary plot of the data shown in (A).

The ability of bulge-inducing conjugates to hybridize with tRNA^Phe^ target was assessed by the electrophoretic mobility shift assay (see Figure 2). Binding of the conjugates to the 3′-FITC-tRNA^Phe^ led to formation of a heteroduplex with lower electrophoretic mobility relative to free 3′-FITC-tRNA^Phe^. By measuring the proportion of the 3′-FITC-tRNA^Phe^ hybridized to the conjugate as a function of the increasing conjugate concentration, the association constants (K_a_) were estimated (see Table 1):

(1) $$\begin{equation*}{\rm{Ka\ }} = \frac{{\rm{\alpha }}}{{{{\left[ {{\rm{BC}}} \right]}_0}\left( {1 - {\rm{\alpha }}} \right)\left( {1 - {\rm{\alpha }}\left( {\frac{{{{[{\rm{tRNA]}}}_0}}}{{{{\left[ {{\rm{BC}}} \right]}_0}}}} \right)} \right)}}\end{equation*}$$

where α is the fraction of RNA bound; [tRNA]_0_ and [BC]_0_ are the total tRNA^Phe^ and conjugate concentrations, respectively.

**Table 1.** Bulge-inducing conjugates: nomenclature, sequence, peptide and association constants (Ka)

Conjugate	Oligonucleotide (5′→3′)	Peptide	K_a_ × 10^6^ M^−1^
**BC2**	TGGTGCGAATT-A*-GTGGATCGAA	[LR]_4_G	0.9 ± 0.3
**BC3**	TGGTGCGAATT-A*-TGGATCGAA	[LR]_4_G	0.5 ± 0.1
**BC4**	TGGTGCGAATT-A*-GGATCGAA	[LR]_4_G	0.4 ± 0.2
**BC5**	TGGTGCGAATT-A*-GATCGAA	[LR]_4_G	0.0042 ± 0.002
**BC2-α/BC2-β**	TGGTGCGAATT-dR-GTGGATCGAACACAG	[LRLRG]_2_	0.9 ± 0.6/0.8 ± 0.3
**BC3-α/BC3-β**	TGGTGCGAATT-dR-TGGATCGAACACAG	[LRLRG]_2_	0.7 ± 0.6/1.0 ± 0.5
**BC4-α/BC4-β**	TGGTGCGAATT-dR-GGATCGAACACAG	[LRLRG]_2_	1.0 ± 0.8/0.9 ± 0.6
**BC5-α/BC5-β**	TGGTGCGAATT-dR-GATCGAACACAG	[LRLRG]_2_	0.4 ± 0.2/0.5 ± 0.2
**BC5L-α/BC5L-β**	TGGTGCGAATT-dR-GATCGAACACAGGAC	[LRLRG]_2_	0.9 ± 0.6/0.5 ± 0.2

All studied conjugates were capable of binding the tRNA^Phe^ in a concentration-dependent manner (Figure 2). The association constants of Type 1 conjugates progressively decreased with a stepwise increase in the size of the induced RNA bulge-loop structures. The conjugate with the longest recognition motif (BC2), which was designed to induce the smallest (2-nt) RNA bulge-loop upon hybridization, showed greater binding affinity toward tRNA^Phe^ than those inducing 3-nt (BC3), 4-nt (BC4) and especially 5-nt (BC5) bulge-loop regions. In fact, K_a_ measured for BC5 under identical conditions (K_a_ = 0.0042±0.002 × 10^6^ M^−1^) was found to be lower by (at least) two orders of magnitude than K_a_ values estimated for BC2, BC3 and BC4 from the same type of conjugates. Such a big drop in binding affinity for BC5 could be attributed to the relatively high stability of the TΨC stem region with three G-C base pairs, none of which was targeted in an antisense fashion by BC5.

Previously, using the 216–312 fragment of influenza virus M2 RNA (M2–96 RNA) (30), we showed that up to seven bulged bases could be tolerated in terms of detectable hybridization. Here, we demonstrate that the significant loss of the binding affinity seen for the 5-nt bulge-loop inducing conjugate (i.e. BC5) can be sufficiently compensated by the elongated recognition motifs. Indeed, the hybridization power of the BC5-α/BC5-β, and especially that of the elongated BC5L-α/BC5L-β conjugates, was re-gained by implementing the longer oligonucleotide recognition motifs, which were designed to cover the entire TΨC arm and even invade the variable loop of the tRNA^Phe^. The association constants of the Type 2 conjugates (BC-α and BC-β series) remained similar (differences in K_a_ did not exceed the statistical errors). The longer recognition motifs used for Type 2 conjugates seem to mask any significant destabilization effects from the induced bulge-loops, which can be easily tolerated if their size is equal or less than 5-nt.

### Cleavage activity against tRNA^Phe^

Cleavage activities of the bulge-loop inducing conjugates were examined against 3′-FITC-tRNA^Phe^ (1 μM) under physiological conditions (37°C, pH 7.0) over 6, 24, 48 and 72 h (see ‘Materials and Methods’ section). 20-fold excess of the conjugate (20 μM) over tRNA was used in the cleavage assays to ensure that even shorter conjugates from Type 1 could achieve the maximum level of binding with the tRNA^Phe^ (Figure 2), although this was impossible to attain for BC5 due to its poor hybridization. Tris buffer (50 mM Tris–HCl pH 7.0, 0.2 M KCl, 1 mM EDTA) was used, because it does not interfere with the RNA transesterification process. The presence of EDTA avoided spontaneous RNA cleavage, which is potentially catalyzed by any traces of (non-univalent) metal ions. Cleavage products were analyzed by electrophoresis in 12% polyacrylamide gel electrophoresis (PAGE) under denaturing conditions and identified by comparison with RNase T1 and 2 M imidazole tRNA^Phe^ hydrolysis ladders.

**Figure 3.**
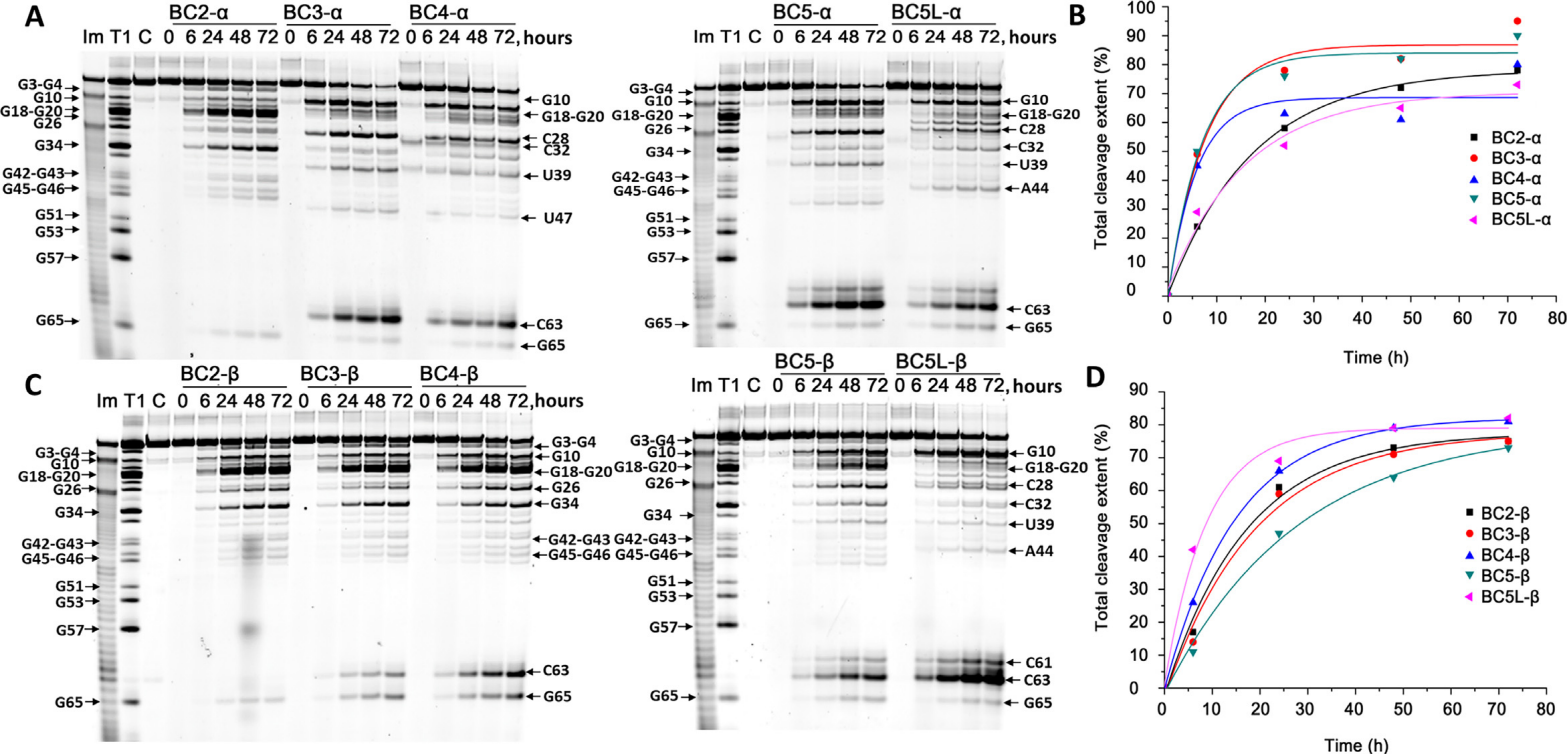
Cleavage of 3′-FITC-tRNA^Phe^ with bulge-forming conjugates BCn-α and BCn-β. Representative images of 12% PAAM/8 M urea gels after electrophoresis of 3′-FITC-tRNA^Phe^ cleavage products with BCn-α (**A**) and BCn-β (**C**) as a function of the incubation time. 3′-FITC- tRNA^Phe^ (1 μM) was incubated with the conjugates (20 μM) in Tris buffer (50 mM Tris–HCl pH 7.0, 0.2 M KCl, 1 mM EDTA) at 37°C. Lanes T1 and Im represent partial 3′-FITC- tRNA^Phe^ digestion with RNase T1 and imidazole, respectively. In lane C, 3′-FITC- tRNA^Phe^ was incubated without conjugates for 72 h. Positions of RNA cleavage by RNase T1 and conjugates are shown on left and right, respectively. Secondary plot of the data indicating the total cleavage extent (%) plotted against the incubation time for BCn-α (**B**) and BCn-β (**D**).

Strikingly, Type 1 bulge-loop inducing conjugates (BC2–BC5) were inactive against tRNA^Phe^ (Supplementary Figure S13), even though all conjugates (except for BC5) were able to fully bind the RNA under these conditions (Figure 2). BC2 showed a very weak cleavage (<5% after 24 h) at U8-A9 site, but outside the target bulge-loop region. In contrast, all Type 2 conjugates (both from BC-α and BC-β series) showed high levels of cleavage activity against the same RNA target (Figure 3), overall reaching 90 and 82%, respectively, for the best structural variants from each series. No spontaneous cleavage of tRNA in the absence of conjugates was seen under these conditions over 72 h (see lanes labeled as C (‘control’) in Figure 3).

**Figure 4.**
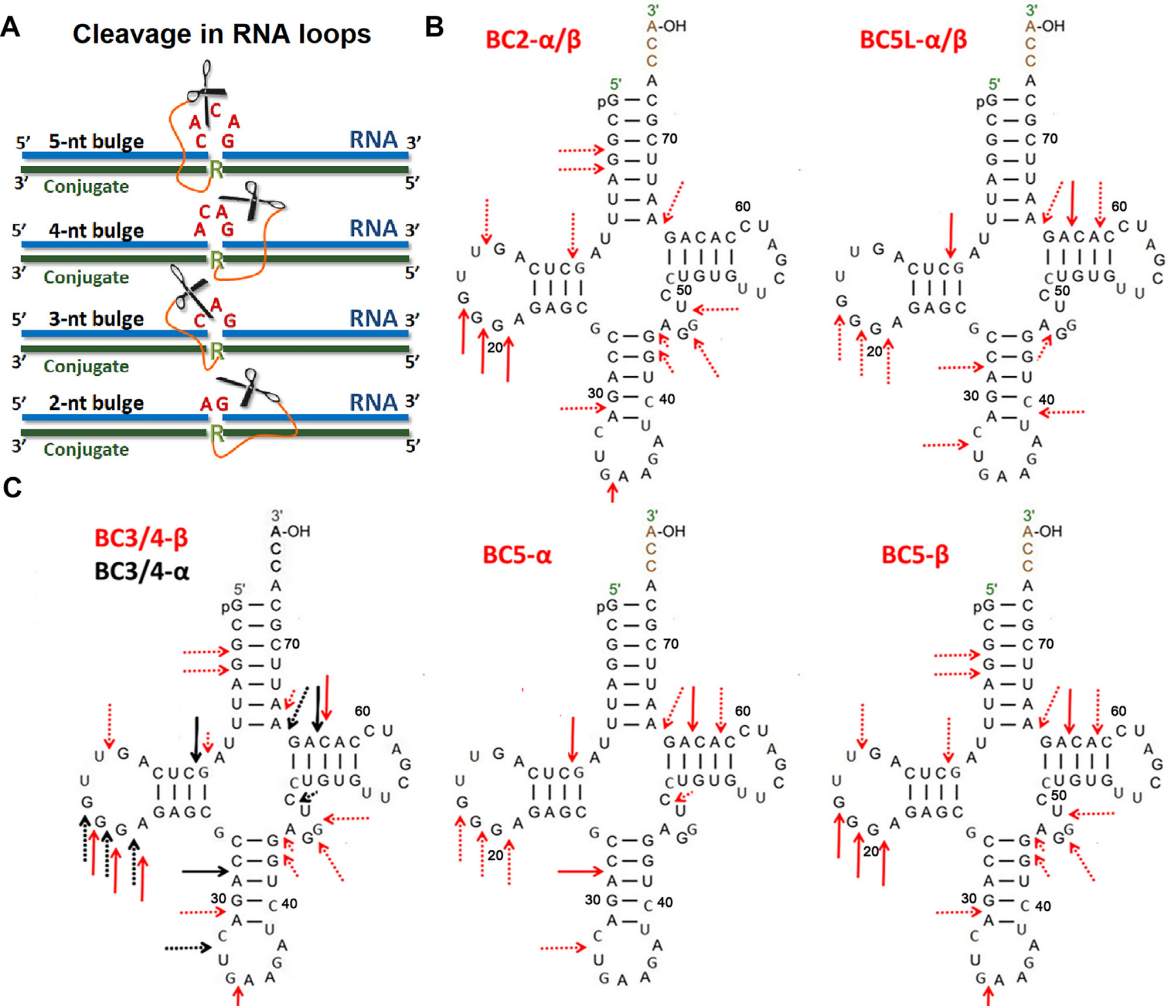
(**A**) Induction of 2-nt, 3-nt, 4-nt or 5-nt bulge-loops in the RNA (blue) upon hybridization with the peptidyl-oligonucleotide conjugates (green), which become susceptible to cleavage by closely juxtaposed peptide (scissors). (**B** and **C**) Cleavage patterns of 3′-FITC-tRNA^Phe^ observed upon treatment with α- and β- series of Type 2 conjugates, summarizing data shown in Figure 3. Arrows indicate the point of cleavage. Major cleavage sites are presented with solid line arrows, whereas minor cleavage sites are indicated with dotted arrows. (**B**) Identical cleavage patterns of 3′-FITC-tRNA^Phe^ were witnessed for BC-2α and BC-2β, as well as for BC5L-α and BC5L-β conjugates. (**C**) Differences in cleavage patterns were witnessed for BC3/4-α conjugates (black arrows) versus BC3/4-β conjugates (red arrows), as well as for BC5-α conjugates against BC5-β conjugates.

Although Type 2 conjugates showed some variations both in cleavage patterns and in cleavage efficiency, which are detailed in Figure 4, there was a clear similarity in their activity against the tRNA^Phe^ with some common trends seen within and between BC-α and BC-β conjugates. First, all Type 2 conjugates catalyzed cleavage within the targeted region ^61^C-^62^A-^63^C-^64^A-^65^G, with efficiency generally related to the size of the induced RNA bulge-loop structure. For example, BC-β conjugates (Figure 3) showed progressive increase in the extent of RNA cleavage at this region from very low (0.5%) for BC2-β, which was expected for the smallest (2-nt) bulge; to 6% (for BC3-β), 16% (for BC4-β) and reaching 19 and 54%, respectively for BC5-β and BCL5-β, which induced the largest (5-nt) bulge-loop in the tRNA^Phe^. Although, in general, the BC-α series demonstrated a similar cleavage trend at the bulge-loop regions, BC4-α and BC5L-α deviated slightly from this trend (Figure 3). This can be related to the α-configuration of the peptide attachment to the oligonucleotide recognition motif, thus presumably reflecting a higher degree of steric hindrance at the conjugation point for BC-α series. This would lead to more frustrated modes of interaction with tRNA^Phe^, which can potentially mask the direct effect from the loop size on cleavage efficiency.

The expansion of the induced bulge-loop afforded the increase in the number of cleaved bonds within the targeted region ^61^C-^62^A-^63^C-^64^A-^65^G. Indeed, the single-stranded 2-nt bulge of BC2-α/β constrained its cleavage to only a single cut at G65-A66. Two cuts were possible from the 3-nt and 4-nt bulge-loop of BC3-α/β and BC4-α/β, respectively. However, the 5-nt bulge-loop of BC5-α/β and BC5L-α/β allowed three cuts in the ^61^C-^62^A-^63^C-^64^A-^65^G region targeted by the recognition motif, with cleavage mainly at the C63-A64 position, and fewer cuts at the G65-A66 and C61-A62 sites.

Another important feature common for all Type 2 conjugates was that, upon sequence-specific hybridization, they were able to attack structural elements of the tRNA^Phe^ located outside the induced bulge-loop region (Figure 3). Indeed, several G-X and Pyr-X sites within the D-arm (e.g. G10), D-loop (e.g. G18-G19-G20-A21), anti-codon stem (e.g. C28-A29) and/or loop (e.g. C32-U33) and variable loop (e.g. U47-C48) were found to be vulnerable for cleavage by this type of conjugates. We have previously reported the ability of the peptidyl-oligonucleotide conjugates to attack distantly located sites within the tertiary tRNA structure upon sequence-specific hybridization. (15,16) Even zero-linker conjugates (15) (P2-A-COOH, P4-A-COOH and P4-A-CONH_2_), with relatively limited conformational freedom, were able to catalyze efficient cleavage at the distant U8-A9 and C72-A73 positions (in addition to their target C63-A64 site), after being hybridized to the TΨC arm and variable loop of the tRNA^Phe^. Furthermore, the ‘dual’ conjugate DC6 with the catalytic peptides located between two recognition motifs (16), targeted to the acceptor stem and the TΨC loop, showed even wider cleavage spectrum by attacking a D-loop (e.g. G18-G19-G20), anti-codon loop (e.g. C34-U35) and a variable loop (e.g. G45-U47) of tRNA^Phe^, in addition to A64-G65 within the closely positioned target region ^61^C-^62^A-^63^C-^64^A-^65^G. In all cases, non-specific cleavage at the distantly located regions was confidently eliminated (15,16) by independent experiments with the 3′-FITC-labeled 96-mer HIV-RNA, which has essentially no homology with yeast tRNA^Phe^. As 3′-FITC-HIV-RNA was unaffected by conjugates under identical conditions (15,16), such cleavage can only occur within the complementary complex with tRNA formed upon sequence-specific hybridization.

The bulge-loop inducing conjugates invented here showed even more diverse and complex cleavage profiles than those reported previously (15,16). This appears to reflect the higher degree of conformational freedom of the catalytic peptide within their hybridized complex with tRNA^Phe^, presumably due to its flexible attachment to the mobile abasic sugar ring (Figure 1). The overall length of the catalytic peptide (45.1 Å), estimated from the maximum size of the unfolded peptide (36.5 Å) measured previously (15) and the length of the aminohexyl linker (8.6 Å), seems to be sufficient to reach most of the sites identified in the tRNA^Phe^ cleavage profiles. Indeed, the distances between the observed cleavage sites and the bridging phosphorus atoms of the ^61^C-^62^A-^63^C-^64^A-^65^G region, opposite to the peptide attachment point, which were evaluated from the available X-ray structure (51) of yeast tRNA^Phe^ as described earlier (16), did not exceed 45 Å. The only exception was the C34-U35 site, which was separated by 54 Å from the peptide conjugation point (16). However, this relatively distant region can potentially be reached by the peptide. When the tertiary structure of tRNA is compromised by partial unfolding of the TΨC loop and acceptor stem upon hybridization with the conjugate, (15,16) the possibility for structural fluctuations make even such distant regions accessible for cleavage. The high degree of conformational freedom of the catalytic peptide, flexibly anchored to the recognition motif of the conjugate, could provide an advantage for potent catalytic destruction of long RNA sequences, especially those with the complex secondary and tertiary structural organization (e.g. long non-coding RNA or viral genomic RNA). Multiple cuts at different locations of the long RNA sequence, induced by a single conjugate molecule upon sequence-specific hybridization, will enhance the potency of the catalytic reaction, and may also increase the turnover effect on potency as well.

The catalytic performance of BC-α conjugates seemed to be slightly superior than that of the BC-β series, extending to 90 and 82% cleavage for the best structural candidates from each category (BC5-α and BC5L-β, respectively). However, the extent of RNA cleavage in loops was higher for the BC-β series as compared to that for the BC-α series (*cf*. 54% versus 45% for BC5L-β and BC5-α, respectively), which might be linked to a higher degree of steric hindrance at the peptide attachment point for α-configuration anomers.

### Enzymatic probing

**Figure 5.**
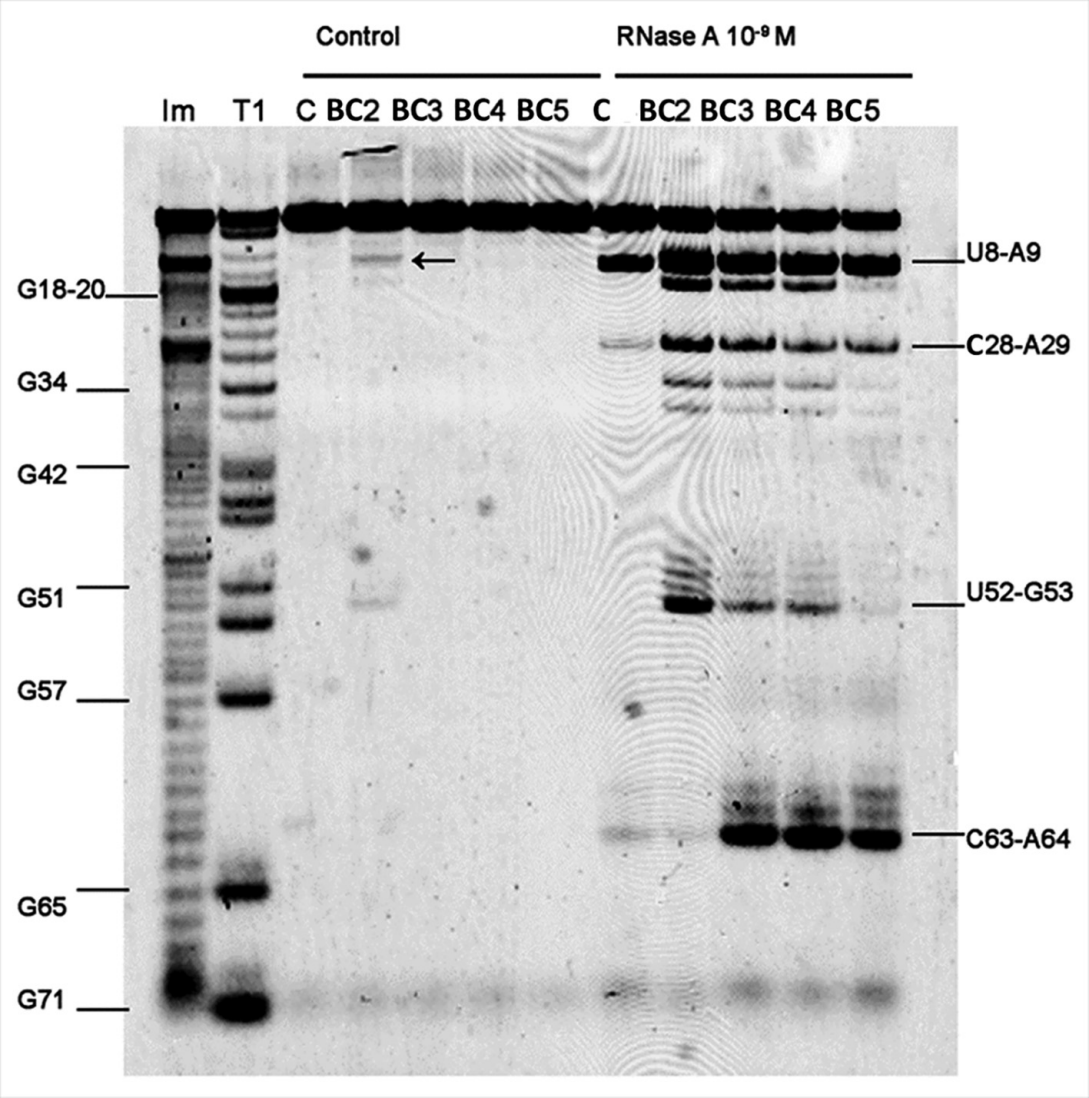
RNase A probing 3′-FITC-tRNA^Phe^ structure within hybridized complexes with the bulge-forming conjugates. Image of 12% PAAM/8 M urea gel electrophoresis: ‘Control’ and ‘RNase A’ at the top indicate samples incubated in the absence and in the presence of RNase A, respectively. In the test experiments, 3′-FITC-tRNA^Phe^ (1 μM) was incubated with one of the Type 1 conjugate (20 μM), for 20 min at 37°C followed by cleavage with RNase A (1 nM) for 10 min at 37°C. Lanes BC2, BC3, BC4, BC5 correspond to complexes of 3′-FITC-tRNA^Phe^ with the respective conjugate. Lane C, 3′-FITC-tRNA^Phe^ incubated without conjugates. Lanes **Im** and **T1**, indicate partial tRNA^Phe^ digestion with 2 M imidazole buffer or RNase T1, respectively. Positions of tRNA^Phe^ digestion with RNases A and T1 are shown on the right and on the left, respectively.

Since none of the Type 1 conjugates (BC2–BC5) were active against tRNA^Phe^, we investigated the possible reasons behind such catalytic failure. First, we examined the structural rearrangements of the tRNA^Phe^ upon hybridization with BC2–BC4 conjugates, using enzymatic probing with RNase A (Figure 5), in order to assess the accessibility of the target RNA regions for cleavage. The molar excess of conjugates over 3′-FITC-tRNA^Phe^ (20:1) was used to achieve the highest possible level of hybridization.

In the absence of conjugates (Figure 5; lane C within the ‘RNase A’ band), RNase A produced one major cut in the tRNA^Phe^ structure at the U8-A9 position and two faint cuts at the C28-A29 and C63-A64 positions. Hybridization of tRNA^Phe^ with BC3, BC4 and BC5 (but not with BC2) led to a significant (>100 times) increase in cleavage at C63-A64 site within the ^61^C-^62^A-^63^C-^64^A-^65^G target region. Presumably, the formation of 3-, 4- and 5-nt containing bulge-loop structures made C63-A64 linkage accessible for cleavage by RNase A. Surprisingly, although BC5 showed poor binding affinity toward 3′-FITC-tRNA^Phe^ (with only 5% of 3′-FITC-tRNA^Phe^ being hybridized with this conjugate under such conditions), it showed increased cleavage at the C63-A64 site. In the presence of BC5, cleavage extended almost to that seen for BC3 and BC4, which showed 100% binding with 3′-FITC-tRNA^Phe^ under these conditions (see Figure 2). This illuminates an important implication that even relatively weak binding affinities of conjugates might be sufficient to promote cleavage of the exposed phosphodiester bonds. An advantage of inducing relatively large bulge-loop structures upon hybridization could be that they provide RNA with sufficient conformational freedom to adopt the *‘in-line’* conformation essential for cleavage.

In contrast, hybridization of BC2 with tRNA^Phe^, which showed the highest binding affinity, did not promote cleavage at the C63-A64 site, because the 2-nt ^64^A-G^65^ bulge-loop formed did not contain any sites sensitive to RNase A cleavage. Interestingly, hybridization of the conjugates with 3′-FITC-tRNA^Phe^ seems to disturb the intact tertiary structure of tRNA, thus leading to the exposure of additional RNA regions and making them accessible for RNase A cleavage. Indeed, all Type 1 conjugates considerably enhanced cleavage by RNase at the U8-A9 and C28-A29 linkages, as compared to that seen for unbound tRNA^Phe^. Furthermore, two additional sites (i.e. C13-A14 and U52-G53) were efficiently cleaved by RNase A when tRNA^Phe^ was hybridized with BC2 > BC3 ≥ BC4, but not with BC5. The extent of tRNA cleavage at these additional sites seems to correlate well with the stability of the hybridized complexes and their K_a_ values (Table 1). The data confirmed our hypothesis that sequence-specific hybridization of such conjugates with large, complex RNA molecules may promote partial distortion of the key elements of their secondary and tertiary structures and expose distant regions for opportunistic cleavage by the catalytic peptide, flexibly anchored to the conjugate recognition motifs.

RNase H cleaves RNA only when it forms a hybrid complex with DNA. As anticipated, the conjugates BC2-BC5 enhanced tRNA^Phe^ cleavage by RNase H in the 3′-acceptor and TΨC regions (Supplementary Figure S14), whereas the size of the ‘silent’ (single stranded) region for cleavage (symptomatic for the bulge-loop formation) correlates well with the expected number of bulged nucleotides (BC5 > BC4 > BC3 > BC2). Catalytic failure of Type 1 conjugates does not appear to be linked to inability to induce single-stranded bulge-loop regions and/or to expose the other secondary and tertiary elements for successful cleavage.

### Molecular dynamics simulations

The structural dissimilarity of Type 1 and Type 2 conjugates could be another reason behind the striking difference in their catalytic performance. Although the peptide length and sequence may also contribute, the most important mechanistically is the two different modes of incorporation of the catalytic peptide into the conjugate structure (Figure 1). Indeed, the well-established mechanism of RNA catalytic cleavage (16,52–56) involves well-coordinated and synchronized action of all key players involved. This is initiated by deprotonation of the 2′-OH group, followed by the formation of a di-anionic pentaoxyphosphorane intermediate and subsequent departure of the 5′-linked nucleotide.(14,52,53) Such a phosphodiester transesterification reaction can be facilitated by various functional groups introduced into the conjugate structure, which can: (i) enhance the rate of deprotonation at the 2′-hydroxyl group, (ii) stabilize the phosphorane intermediate and (iii) the leaving group and/or (iv) promote the *‘in-line’* geometry, when the 2′-oxyanion enters (or the 5′-linked nucleotide leaves) the phosphorane intermediate.(53,54) In such an *‘in-line’* configuration, which is crucial for catalysis, the penta-coordinate intermediates assume trigonal bipyramidal geometry, with the 2′ and 5′ oxygens occupying apical positions around 180° from one to another (52). Arginine guanidinium groups of the catalytic peptide (acetyl-[LR]_4_G-CO_2_H or acetyl-[LRLRG]_2_-CO_2_H) are key in promoting catalysis (15,16). They appear to act as proton shuttles (53) through various tautomeric forms, by facilitating the proton transfer between the attacking 2′-OH, non-bridging phosphate oxygen and departing 5′-O group. A few reports (55–57) provide evidence of synchronized action of two guanidinium groups in catalysis, when they are present in the same molecular structure. It is postulated that catalysis by such a guanidine-guanidinium dyad is achieved through cooperative proton transfer between the neutral and protonated guanidinium residues. A neutral guanidine acts as a general base to deprotonate the hydroxyl group, while the protonated guanidinium moiety plays a dual role in catalysis by: (i) coordinating the phosphate group, by electrostatic interaction; and (ii) acting as an electrophilic activator, facilitating the transesterification reaction.

**Figure 6.**
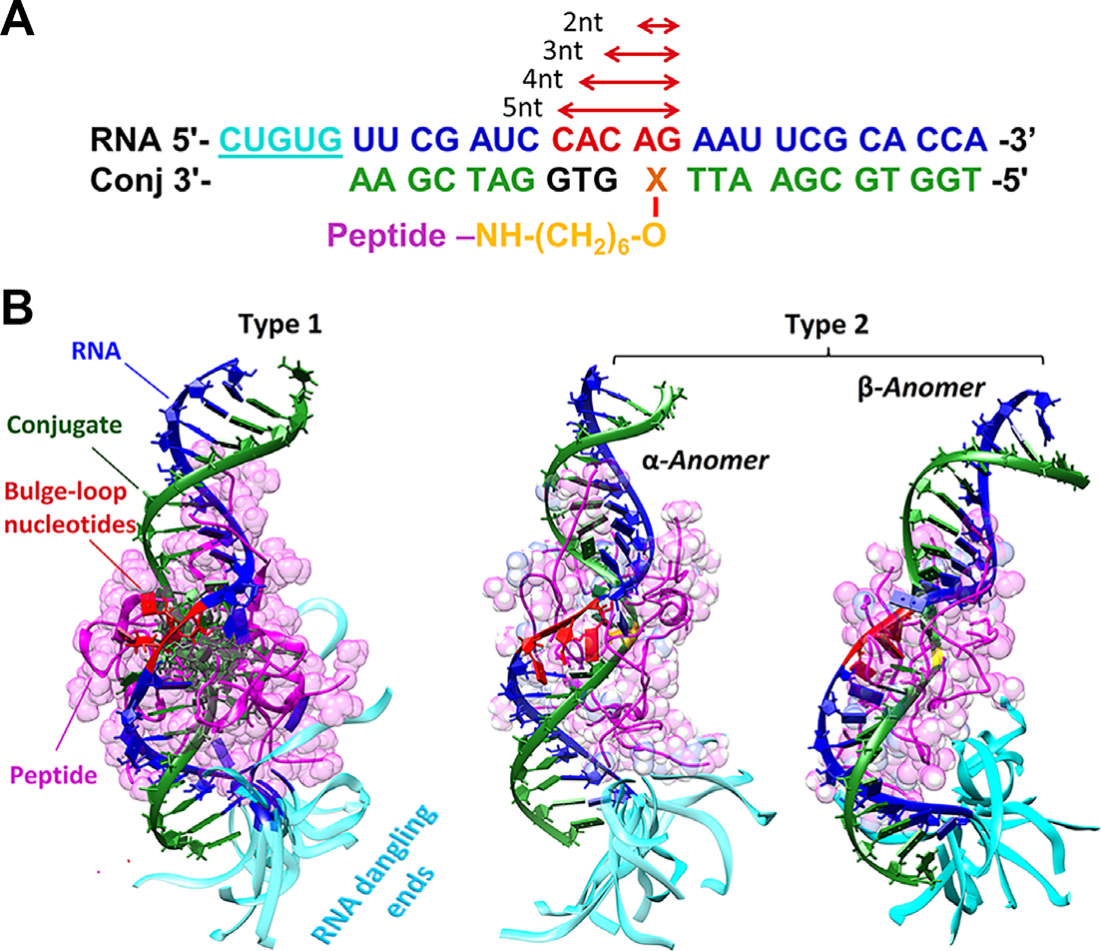
(**A**) Model systems used for the MD simulations. The RNA sequences (blue) replicate the 3′-acceptor stem and the TΨC-loop of the yeast tRNA^Phe^ with an additional dangling end (underlined single-stranded sequence; cyan). The bulge-loop regions of different sizes ranging from 2-nt to 5-nt are highlighted in red. The representative conjugate sequences are shown in green, with the variable region (i.e. truncated for the 3-, 5- and 5-nt bulge-loop inducing conjugates) shown in black. **X** represents the point of attachment of the peptide (either Acetyl-[LR]_4_G-CO_2_H or Acetyl-[LRLRG]_2_-CO_2_H) to either modified adenosine residue (Type 1 conjugates) or abasic nucleotide (Type 2 conjugates from BC-α and BC-β series). The linker and peptide are indicated in yellow and pink, respectively. (**B**) Cluster analysis of a series of 100 MD simulations of the *RNA-Conjugate* heteroduplexes, using SA. The RNA fragment (blue) was hybridized with the bulge-loop conjugate (green) either from Type 1 BC3 (**left**), or Type 2 series (BC3-α (**center**) or BC3-β (**right**)). The peptide backbone and sidechains are shown in pink, bulge loop regions in red, and the single-stranded 5′-dangling ends in cyan. The corresponding rotating images of the structures are given in the Supplementary Data.

Given a strict structural demand for all functional groups involved in catalysis, and the necessity for the RNA cleavage sites to attain an *‘in-line’* geometry to promote transesterification, we used MD simulations. 12 *RNA*-*Conjugate* hybrids model systems were generated, four from each category (4 × Type 1 conjugates including BC2-BC5, and 8 × Type 2 conjugates including 4 × BC-α and 4 × BC-β conjugates), each interacting with the RNA fragment replicating the relevant part of the yeast tRNA^Phe^ sequence with an elongated 5′-dangling end (Figure 6A). We maintained the same sequence and length of the oligonucleotide for each type of bulge-loop category (i.e. 2-nt, 3-nt, 4-nt or 5-nt), which was identical to the recognition motif of BC2, BC3, BC4 or BC5 conjugates (Figure 6A). Before the 10 ns productive simulation, each model system was subjected to a series of 100 SA runs in implicit solvent, involving heating to 800 K for 0.5 ns, followed by an equilibration at 800 K for 5 ns and cooling down to 300 K over 0.5 ns, with subsequent equilibration at 300 K over another 5 ns (see ‘Materials and Methods’ section for details). Cluster analysis of 10 ns productive simulation (Figure 6B) showed that all calculated *DNA:RNA* hybrids adopted the intermediate conformation between A and B forms of a double-stranded nucleic acid. The introduction of the bulge-loops caused bending of the overall structure, as earlier reported for *DNA:DNA* duplexes, but without significant change of the global conformation (58). Such a *RNA-Conjugate* complex can potentially be recognized by cellular RNase H, thus boosted by a synergetic effect from the joint action of conjugates and RNase H, as we recently demonstrated experimentally (20).

**Figure 7.**
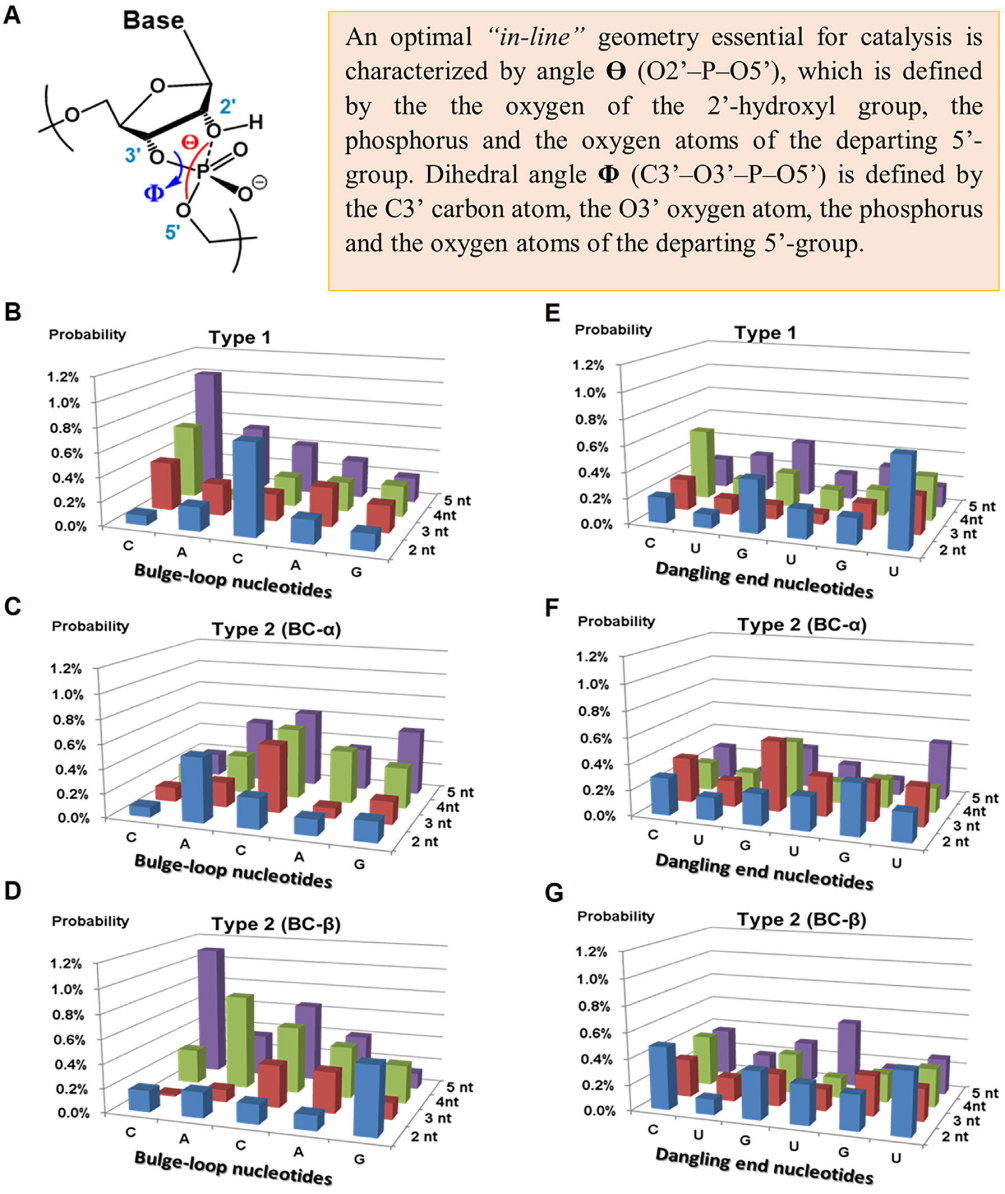
(**A**) An optimal *‘in-line’* geometry (59) essential to promote catalyzed transesterification in RNA sequences. (**B**–**D**) The probability of *‘in-line’* conformation evaluated for the RNA nucleotides within the bulge-loop regions of the complexes formed between RNA and Type 1 conjugates (**B**) and Type 2 conjugates from BC-α (**C**) and BC-β (**D**) series. (**E** and **F**) The probability of *‘in-line’* geometry evaluated for the RNA nucleotides from the dangling ends of the complexes formed between RNA and Type 1 conjugates (**E**) and Type 2 conjugates from BC-α (**F**) and BC-β (**G**) series. The probabilities of achieving an *‘in-line’* geometry optimal for catalysis, estimated from the favorable values of **Θ** (when **Θ** >155°), which can be achieved during the MD simulation, were calculated as the ratio between the number of structures with **Θ** > 155° in MD trajectory to the overall number of all structures.

For each hybrid complex, we have estimated a propensity for RNA cleavage via *‘in-line attack’* based on the measured angle **Θ** (O2′–P–O5′) of the RNA nucleotides from the bulge-loop-region (59). The realization of the *‘in-line attack’* conformation to promote catalysis can be achieved by satisfying a specific range of values for the dihedral angle **Φ** (-25≤ **Φ** ≤ 10°), which strongly correlates to the region of the optimal *‘in-line’* geometry (59) defined by angle **Θ**, when **Θ** ≥155° (Figure 7). We found that relatively favorable values of **Θ** could potentially be achieved by most of the nucleotides from the bulge-loop region ^61^C-^62^A-^63^C-^64^A-^65^G, when RNA is hybridized with either Type 1 or Type 2 conjugates, regardless of their category. Instead, the probability of adopting *‘in-line’* geometry seemed to correlate strongly with the length of the induced RNA bulge-loop region, with the clear preference of the larger loop size in the order 5-nt >4-nt >3-nt >2-nt (Figure 7B–D).

**Figure 8.**
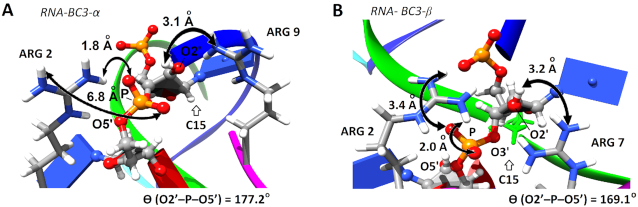
The expanded regions of the selected hybrid structures *RNA-BC3-α* (**A**) and *RNA- BC3-β* (**B**) showing a realization of an *‘in-line attack’* conformation with **Θ** (O2′-P-O5′) ≥155°. The guanidinium groups of the two arginine residues are located close to the sugar-phosphate backbone of the bulged nucleotide C15 to form a *guanidine-guanidinium* dyad, essential for catalysis.

Although in some cases 2-nt bulge-loop inducing conjugates may allow adoption of *‘in-line’* geometry for A62, C63 and/or G65 nucleotides, most of these residues (except for G65) are involved in duplex formation, which seems to protect these sites from cleavage. Interestingly, most of the nucleotides from the RNA single-stranded dangling ends (Figure 7E–G) showed the probabilities for achieving an *‘in-line’* geometry similar to those in loops, thus suggesting that such flexible single-stranded RNA regions are inherently vulnerable for cleavage and ready for an opportunistic attack from closely located catalytic groups. This agrees with our experimental data (Figures 3 and 4), showing that the single-stranded regions from D-loop (e.g. G18-G19-G20-A21), anti-codon loop (e.g. C32-U33) and variable loop (e.g. U47-C48) could be cleaved by all Type 2 conjugates, irrespective to their length. The expanded regions of the selected hybrid structures RNA-BC3-α and RNA-BC3-β showing a realization of an ‘in-line attack’ conformation are given in Figure 8 (see also the corresponding rotating images in the Supplementary Data).

The cluster analysis showed that in all hybrid structures the positively charged peptide was engaged in strong interactions with the negatively charged sugar-phosphate backbone of the nucleic acids through electrostatic molecular forces (see Figure 6 and the corresponding rotating images in the Supplementary Information). These contacts included the interactions with the single-stranded bulge-loop sites (red) opposite to the peptide conjugation point, the double-stranded *RNA–DNA* regions, and even with the RNA dangling ends, all of which could potentially be destructive for catalysis. This is consistent with our experimental data (see Figures 3 and 4) showing that Type 2 conjugates (BC-α and BC-β) cleaved RNA at distantly located sites, in addition to the target bulge-loop region.

Finally, we have analyzed the ability of the catalytic guanidinium groups of the four arginine residues in BC3, BC3-α and BC3-β to reach the RNA bulge-loop region, by evaluating the distribution of distances between the nitrogen atoms of each guanidinium group and O2′ and/or phosphorus atoms of every ‘bulged’ nucleotide (Supplementary Figure S15). Based on the suggested mechanism of the guanidinium-catalyzed transesterification, (55–57) we assumed that, within the same molecular framework, a prerequisite for successful cleavage is the simultaneous presence of all these atoms at short distances, not exceeding 6 Å.

We discovered (Supplementary Figure S15) that, although there is a high probability of finding the catalytic guanidinium groups at locations distant from the sugar-phosphate backbone of the bulge-loops (i.e. between 6 Å and 43 Å), there was a certain proportion of structures, where the key atomic players were closely located at distances ≤6 Å (see Figure 8 and Supplementary Figure S15B, as well as the corresponding rotating images in the Supplementary Data). Crucially, the peptide attachment through a flexible abasic sugar residue (BC3-α and BC3-β from Type 2 series) led to ∼3-fold higher contribution from the short-range distance distribution (Supplementary Figure S15B) for these atoms as compared to Type 1 series. As anticipated, the presence of an additional glycine residue in the longer peptide acetyl-[LRLRG]_2_-CO_2_H in Type 2 conjugates enhanced the overall flexibility of its catalytic function, allowing exploration of more energetically favorable conformations, which is in agreement with the high activity of the Type 2 conjugates. Importantly, the nuclease-resistant α-anomer, which offers an advantage of potentially superior cellular stability of the conjugates *in vivo*, also showed a slightly more favorable setting for cleavage as compared with β-anomer (Supplementary Figure S15B). Overall, in the hybrid complexes formed by Type 1 conjugates, the catalytic peptide was located more distantly from the RNA bulge-loop region (Supplementary Figure S15A), with the weighted average distance of 20 Å between the catalytic guanidinium groups and O2′ and/or phosphorus atoms of the ‘bulged’ nucleotides (*cf*. with 17 Å and 18 Å for BC3-α and BC3-β, respectively). The adenosine residue was the peptide attachment point for Type 1 and tends to adopt more energetically favored *anti* conformation, thus restricting the ability of the peptide to approach the RNA bulge-loop region and exert its function.

## CONCLUSION

Of the library of 14 bulge-loop inducing peptidyl-oligonucleotide conjugates, developed here for catalytic sequence-specific destruction of RNA sequences, two very distinct types of behavior arose. Type 1, exemplified by conjugation of catalytic peptide via an aminohexyl-modified adenosine, showed zero activity against RNA. In contrast, Type 2, achieved by conjugation of the longer catalytic peptide via an internal abasic nucleotide, whether in α- or β-configuration, showed up to 90% total RNA cleavage. This striking difference can be related to structural requirements of catalysis, also illuminated by MD simulations of these bulge-loop inducing conjugates: (i) flexible attachment of the catalytic peptide via an internal abasic nucleotide either in α- or β-configuration; (ii) avoidance of peptide incorporation via the aromatic nucleotide base, to minimize any conformational restraints; (iii) induction of ≥3-nt RNA bulge-loop regions to provide an opportunity for RNA to adopt *‘in-line’* conformation; and (iv) incorporation of a longer peptide with an additional glycine in the middle to enhance structural flexibility. Our insights into the structural organization of the hybrid complexes between the conjugates and RNA, and their MD simulation can now guide design to optimize interactions vital for catalysis, particularly (i) to minimize the probability of ‘non-productive’ conformations and (ii) to achieve multiple catalytic turnover by each conjugate molecule, which is essential for therapeutic destruction of pathogenic cellular RNA.

In order to allow these discoveries to progress into translational success, it will be crucial in the future to achieve sufficient serum stability of such bioconjugates *in vivo*. Although the peptide provides the oligonucleotide with some level of protection from cellular nucleases, (17,20) yet more biologically stable variants can be produced by harnessing recent advances in nuclease-resistant chemical modification, (60,61) which may also enhance binding to the target RNA. Indeed, the newly discovered triazole-LNA and carbamate-LNA modified oligonucleotides (62,63) show a much reduced level of degradation *in vivo* and significantly higher affinity toward RNA/DNA, which is re-enforced by locked nucleic acid sugars, thus providing us with new opportunities. This might allow the use of shorter oligonucleotides with improved cellular uptake properties, especially when conjugated to the arginine-rich catalytic peptides, which have been previously shown to facilitate transport of large hydrophilic molecules across biological barriers (64–68).

## Supplementary Material

gkaa780_Supplemental_Files

## Notes

Present address: Aled Williams, AstraZeneca Pharmaceutical Technology and Development, Macclesfield, SK10 2NA, UK.
