# Supplementary material for: Strict conformational demands of RNA cleavage in bulge-loops created by peptidyl-oligonucleotide conjugates
Source: Nucleic Acids Res. 2020 Oct 3;48(19):10662–79. doi: 10.1093/nar/gkaa780 (PMC7641753; doi:10.1093/nar/gkaa780)
Supplement: gkaa780_Supplemental_Files [file gkaa780_supplemental_files.zip › NAR supplementary final version.pdf]

Strict conformational demands of RNA cleavage in bulge-loops created by peptidyl-oligonucleotide conjugates

Yaroslav Staroseletz[1,†], Bahareh Amirloo[2,†] , Aled Williams[2], Alexander Lomzov[1], Kepa K. Burusco[2], David J. Clarke[2], Tom Brown[3], Marina A. Zenkova[1] and Elena V. Bichenkova[2,*]

[1] Institute of Chemical Biology and Fundamental Medicine SB RAS, 8 Laurentiev Avenue, 630090, Novosibirsk, RF

[2] School of Health Sciences, Faculty of Biology, Medicine and Health, University of Manchester, Oxford Road, Manchester, M13 9PT, UK

[3] Department of Chemistry, Chemistry Research Laboratory, University of Oxford, 12 Mansfield Road, Oxford, OX1 3TA, UK

SUPPORTING INFORMATION

Table of contents

Sequences of the unconjugated oligonucleotides and peptides **2**

1. **Figure S1. Design concept for Type 1 bulge-loop inducing conjugates (BC2 - BC5)** **3**
2. **Figure S2. Design concept for Type 2 bulge-loop inducing conjugates BC-α and BC-β** **4**
3. **Figure S3. Shift in RP-HPLC purification chromatogram for Type 1 conjugates (BC2-BC5)** **5**
4. **Figure S4. Shift in RP-HPLC purification chromatogram for Type 2 conjugates** **5**
5. **Figure S5. ESI-MS spectra of Acetyl-$[LR]_4G$-CO_2H peptide** **6**
6. **Figure S6. ^{1}H-NMR spectra of the Acetyl-$[LR]_4G$-CO_2H peptide** **6**
7. **Figure S7. MALDI-ToF mass spectra Acetyl-$[LRLRG]_2$-CO_2H** **7**
8. **Figure S8. ^{1}H-NMR spectra of the Acetyl-$[LRLRG]_2$-CO_2H peptide** **7**
9. **Figure S9. MALDI-ToF mass spectra of the peptidyl-oligonucleotide conjugates BC2-BC5** **8**
10. **Figure S10. ^{1}H NMR spectra of the bulge-loop inducing conjugates BC2-BC5** **9**
11. **Figure S11. MALDI-ToF mass spectra of the peptidyl-oligonucleotide conjugates BC-α and BC-β** **10**
12. **Figure S12. ^{1}H NMR spectra of the bulge-loop inducing conjugates BC-α and BC-β** **12**
13. **Figure S13. Cleavage of 3'-FITC-tRNA^{Phe} with Type 1 conjugates (BC2-BC5)** **14**
14. **Figure S14. RNase H cleavage assay** **14**
15. **Figure S15. Distance distributions between guanidinium group of arginines and O2' or phosphorous atoms of the bulged nucleotides of RNA-conjugate duplex** **15**
16. **ZIP files** **15**

---

† These authors contributed equally to this work.

* Corresponding Author

Sequences of the unconjugated oligonucleotides and peptides

Table S1. Sequences of the unconjugated oligonucleotides and peptides used for conjugate reactions along with their millimolar extinction coefficients.

**Name**	**Sequence (5' to 3')**	**E_{260} ($mM^{-1}\cdot cm^{-1}$)[a]**
**Acetyl-[LR]$_4$G-CO_2H**	Ac-Leu-Arg-Leu-Arg-Leu-Arg-Leu-Arg-Gly	n/a
**Acetyl-[LRLRG]$_2$-CO_2H**	Ac-Leu-Arg-Leu-Arg-Gly-Leu-Arg-Leu-Arg-Gly	n/a
**BC2**	TGGTGCGAATT**A***GTGGATCGAA [b]	226.3
**BC3**	TGGTGCGAATT**A***TGGATCGAA [b]	215.9
**BC4**	TGGTGCGAATT**A***GGATCGAA [b]	208.1
**BC5**	TGGTGCGAATT**A***GATCGAA [b]	197.7
**BC2-α**	TGGTGCGAATT-**dR^{alpha}**-GTGGATCGAACACAG [c]	266.7
**BC3-α**	TGGTGCGAATT-**dR^{alpha}**-TGGATCGAACACAG [c]	256.3
**BC4-α**	TGGTGCGAATT-**dR^{alpha}**-GGATCGAACACAG [c]	248.3
**BC5-α**	TGGTGCGAATT-**dR^{alpha}**-GATCGAACACAG [c]	237.9
**BC5L-α**	TGGTGCGAATT- **dR^{alpha}**-GATCGAACACAGGAC [c]	269.1
**BC2-β**	TGGTGCGAATT-**dR^{beta}**-GTGGATCGAACACAG [c]	266.7
**BC3-β**	TGGTGCGAATT-**dR^{beta}**-TGGATCGAACACAG [c]	256.3
**BC4-β**	TGGTGCGAATT-**dR^{beta}**-GGATCGAACACAG [c]	248.3
**BC5-β**	TGGTGCGAATT-**dR^{beta}**-GATCGAACACAG [c]	237.9
**BC5L-β**	TGGTGCGAATT- **dR^{alpha}**-GATCGAACACAGGAC [c]	269.1

a) **E_{260} denotes the millimolar extinction coefficient**

b) **A* denotes aminohexyl-modified adenosine residue**

c) **dR denotes abasic nucleotide incorporated in oligonucleotide seqeucne**

1. Figure S1. Design concept for Type 1 bulge-loop inducing conjugates (BC2 - BC5)

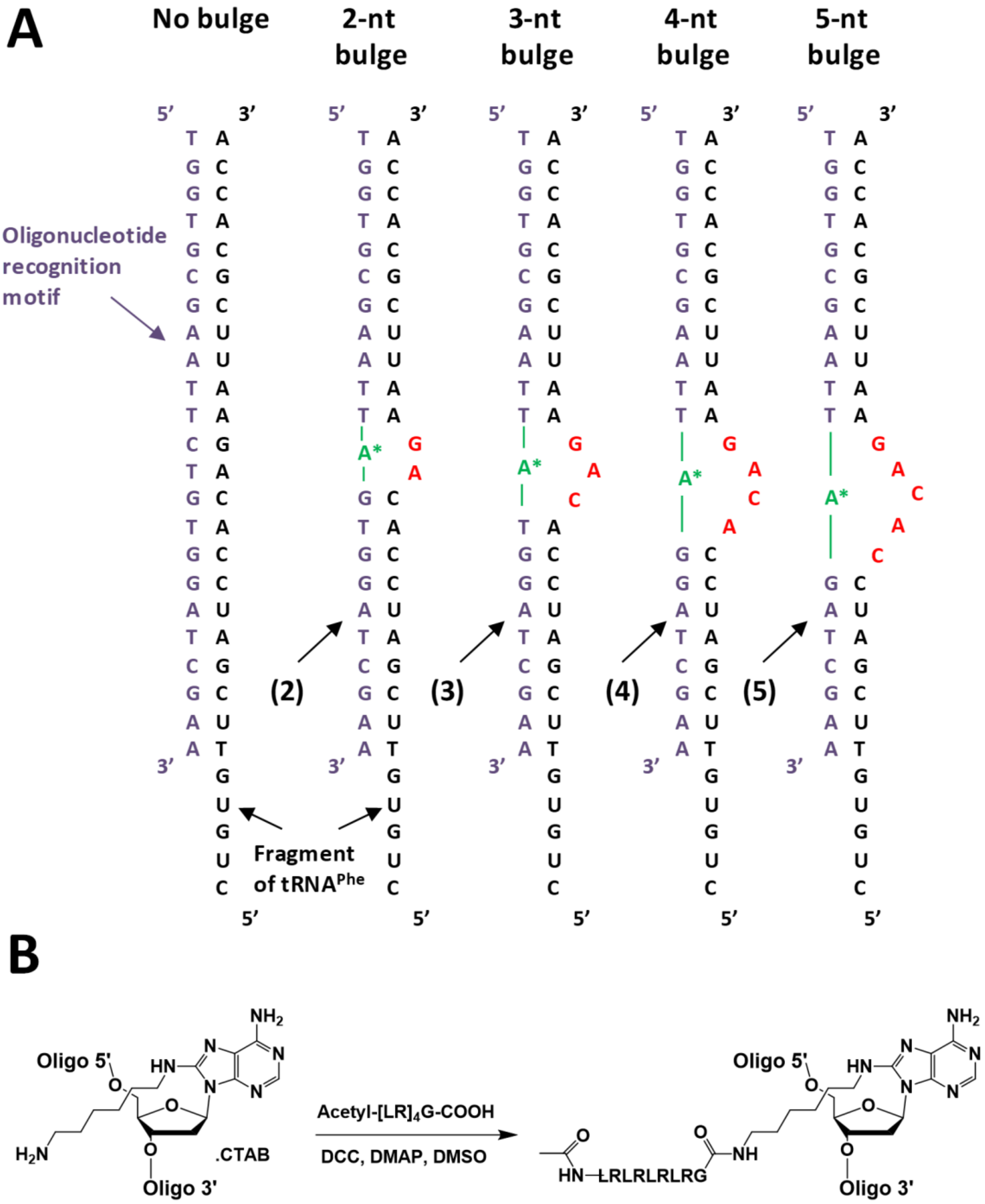

Figure S1. A. Schematic representation of the design concept for the Type 1 bulge-loop inducing conjugates. Oligonucleotides **2**, **3**, **4** and **5** (indicated here by arrows) were used as the recognition motifs for the peptidyl-oligonucleotide conjugates BC2, BC3, BC4 and BC5, respectively. **B.** Synthetic route for the conjugation. Chemical structure of the aminohexyl modified adenosine residue **A*** is indicated along with the oligonucleotide and peptide sequences. To avoid peptide self-conjugation and cyclisation during amide coupling reaction, acetylated N-termini peptides were used.

2. Figure S2. Design concept for Type 2 bulge-loop inducing conjugates BC-α and BC-β

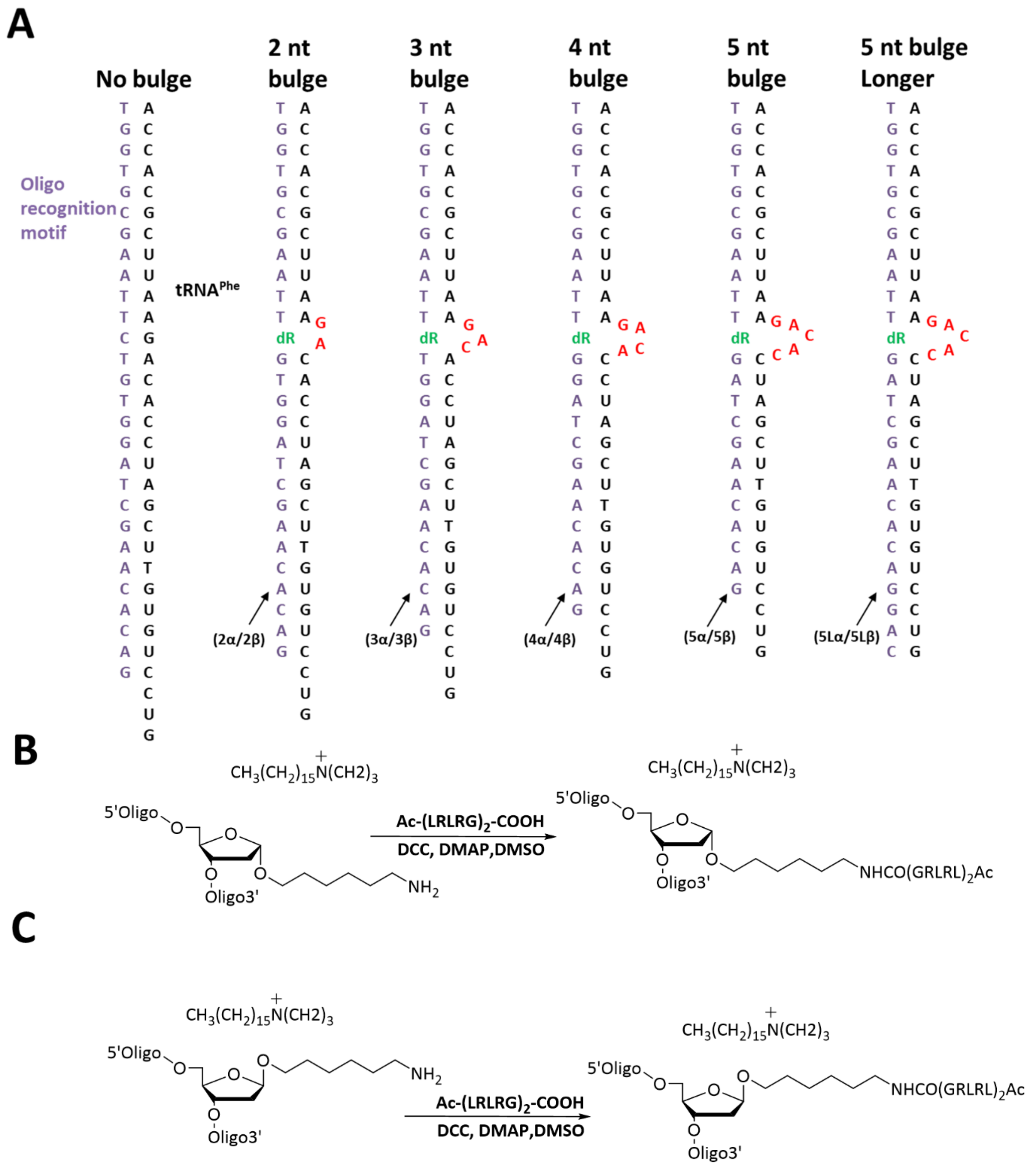

Figure S2. A. Schematic representation of the design concept for the α- and β- anomers of Type 2 bulge-loop inducing conjugates. Oligonucleotides **2α/2β**, **3α/3β**, **4α/4β**, **5α/5β** and **5Lα/5Lβ** (indicated by arrows) were used as the recognition motifs for conjugates BC2-α/ BC2-β, BC3-α/ BC3-β, BC4-α/ BC4-β, BC5-α/ BC5-β and BC5L-α/BC5L-β, respectively. **B.** Synthetic route for the preparation of α-series of the conjugates. **C.** Synthetic route for the conjugation of β-series of the conjugates. Chemical structure of the abasic nucleotide with aminohexyl linker attached at C1’ position **dR** is indicated along with the oligonucleotide and peptide sequences. To avoid peptide self-conjugation and cyclisation during amide coupling reaction, acetylated N-termini peptides were used.

3. Figure S3. Shift in RP-HPLC purification chromatogram for Type 1 conjugates (BC2-BC5)

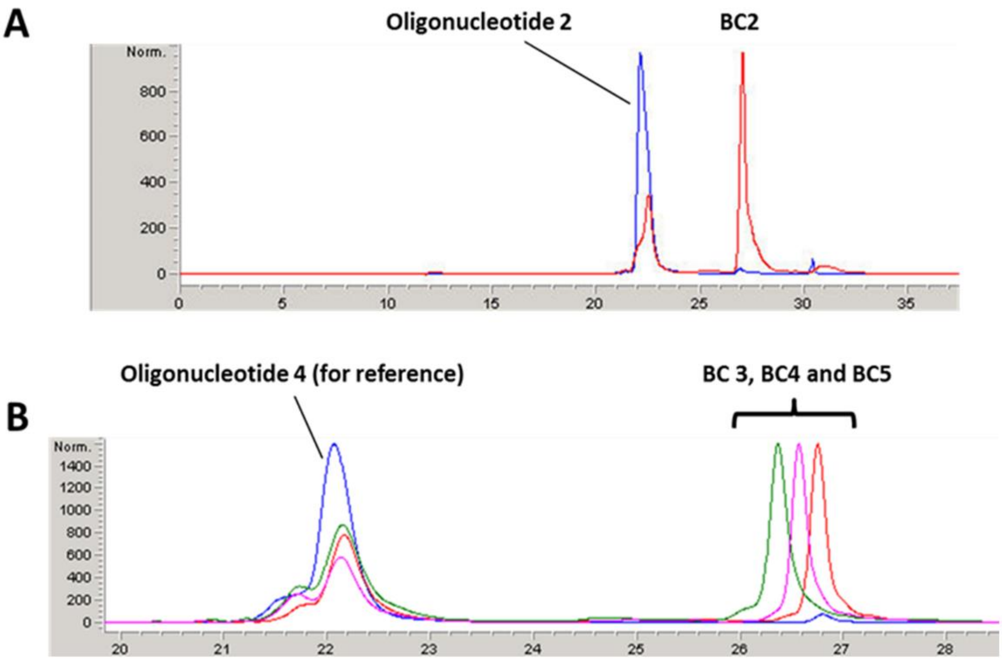

Figure S3. A. An overlay of the RP-HPLC purification chromatograms showing the shift in retention of BC2 (red) compared to unmodified oligonucleotide 2 (blue). **B.** An overlay of the RP-HPLC purification chromatograms of BC3 (green), BC4 (pink) and BC5 (red) showing retention time shifts compared to unmodified oligonucleotide 4 (blue), as a reference.

4. Figure S4. Shift in RP-HPLC purification chromatogram for Type 2 conjugates

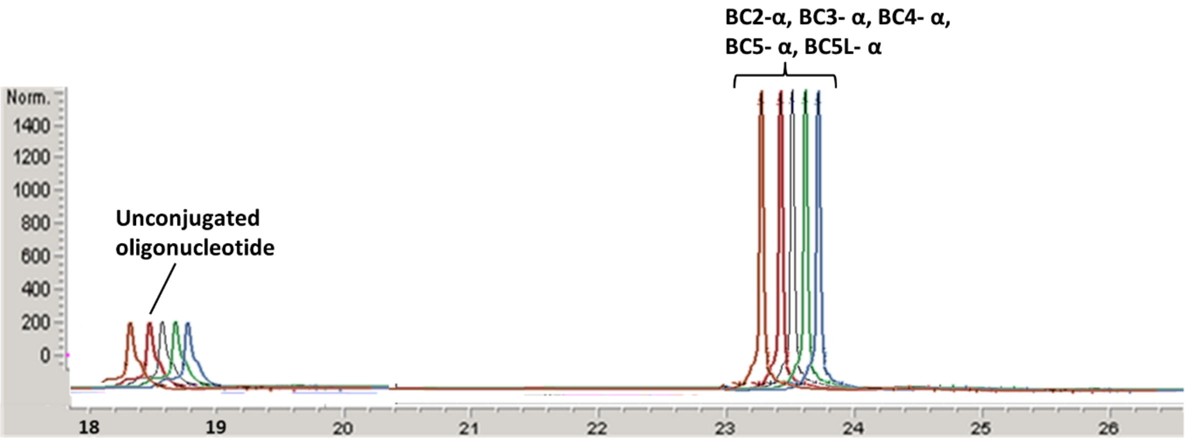

Figure S4. An overlay of the RP-HPLC purification chromatogram of BC2-α (orange), BC3-α (red), BC4-α (black), BC5-α (green), BC5L-α (blue) showing the shift in retention from unconjugated oligonucleotide to conjugate. The same shift in retention time was witnessed during RP-HPLC purification of β-series of the conjugates.

5. Figure S5. ESI-MS spectra of acetyl-[LR]$_4$G-CO_2H peptide

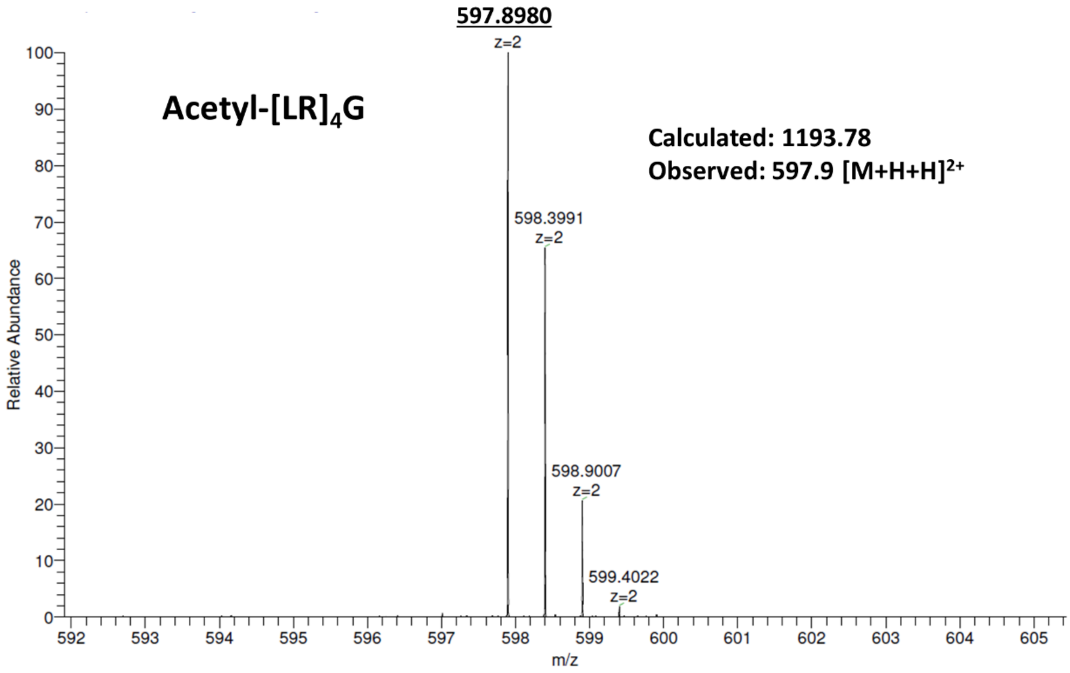

Figure S5. ESI-MS mass spectra of acetyl-[LR]$_4$G peptide. The spectrum was recorded by a Thermo Scientific LTQ Orbitrap XL mass spectrometer.

6. Figure S6. ^{1}H-NMR spectra of the acetyl-[LR]$_4$G-CO_2H peptide

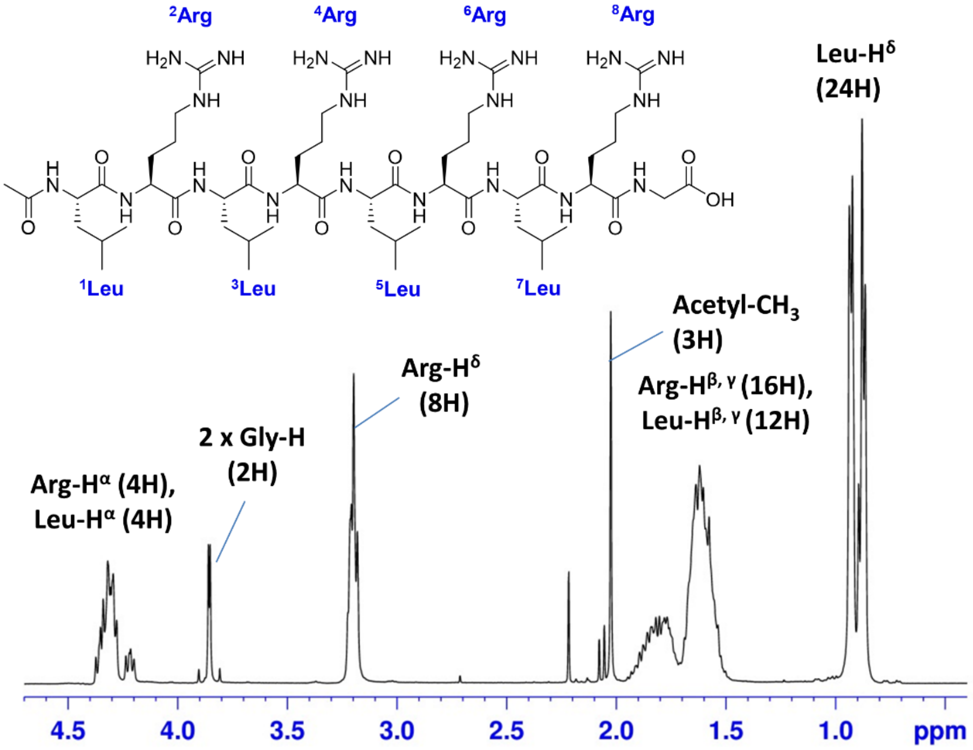

Figure S6. ^{1}H-NMR spectra of the peptide acetyl-[LR]$_4$G-CO_2H. The spectrum was recorded in D_2O at 25 °C using a 400 MHz NMR spectrometer (Bruker Avance II+ 400).

7. Figure S7. MALDI-ToF mass spectrum of acetyl-[LRLRG]$_2$-CO_2H

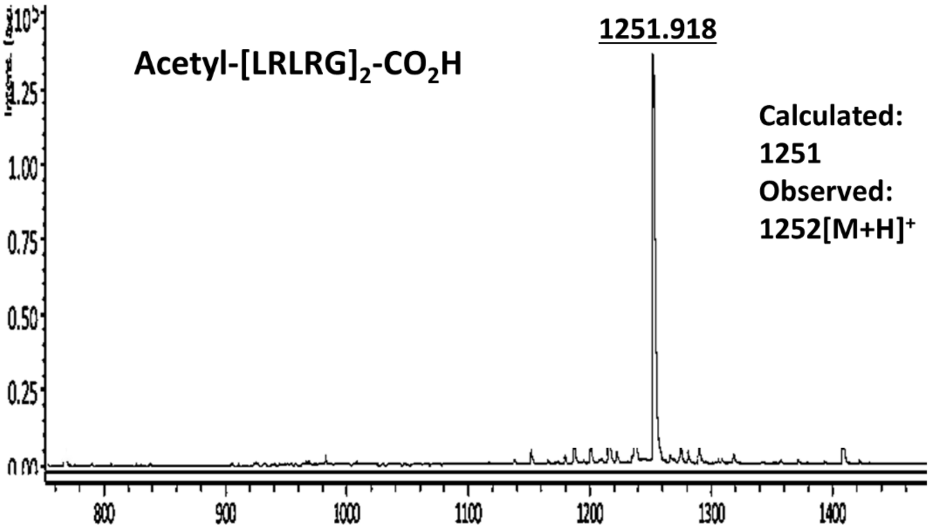

Figure S7. MALDI-TOF spectrum of peptide acetyl-[LRLRG]$_2$-CO_2H. Spectra were recorded using a Bruker Daltonics Ultraflex TOF/TOF mass spectrometer.

8. Figure S8. ^{1}H-NMR spectrum of acetyl-[LRLRG]$_2$-CO_2H peptide

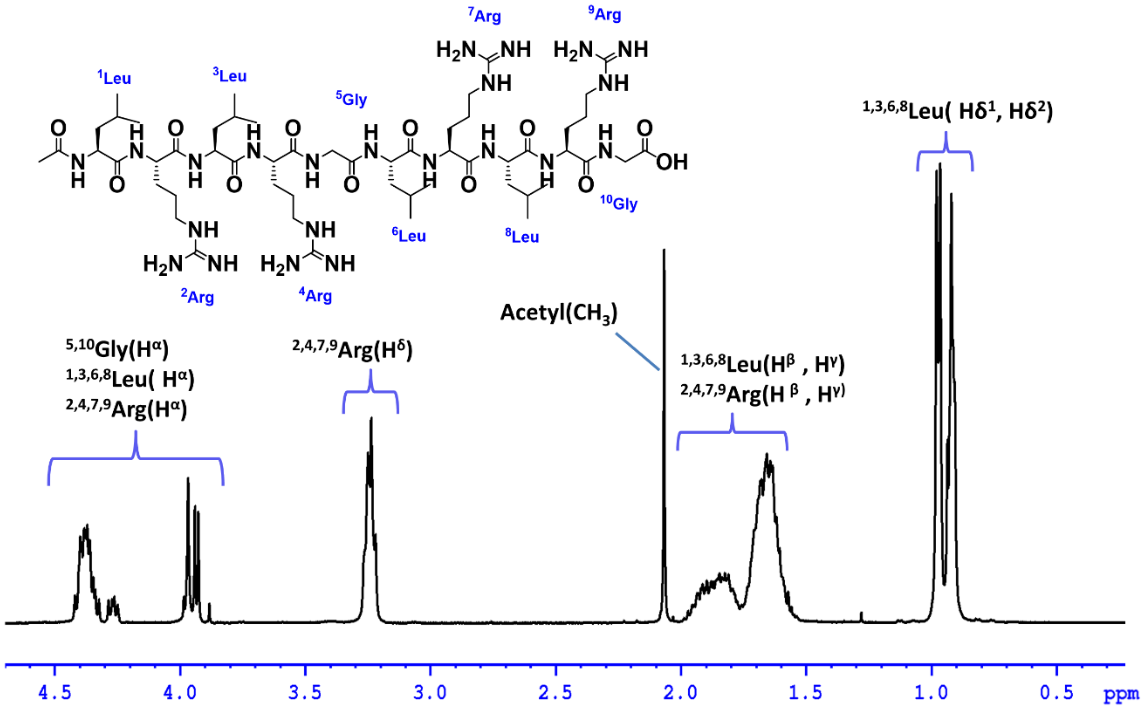

Figure S8. ^{1}H NMR spectrum for acetyl-[LRLRG]$_2$-CO_2H peptide. The spectrum was recorded in D_2O at 25 °C using a 400 MHz NMR spectrometer (Bruker Avance II+ 400).

9. Figure S9. MALDI-ToF mass spectra of the peptidyl-oligonucleotide conjugates BC2-BC5

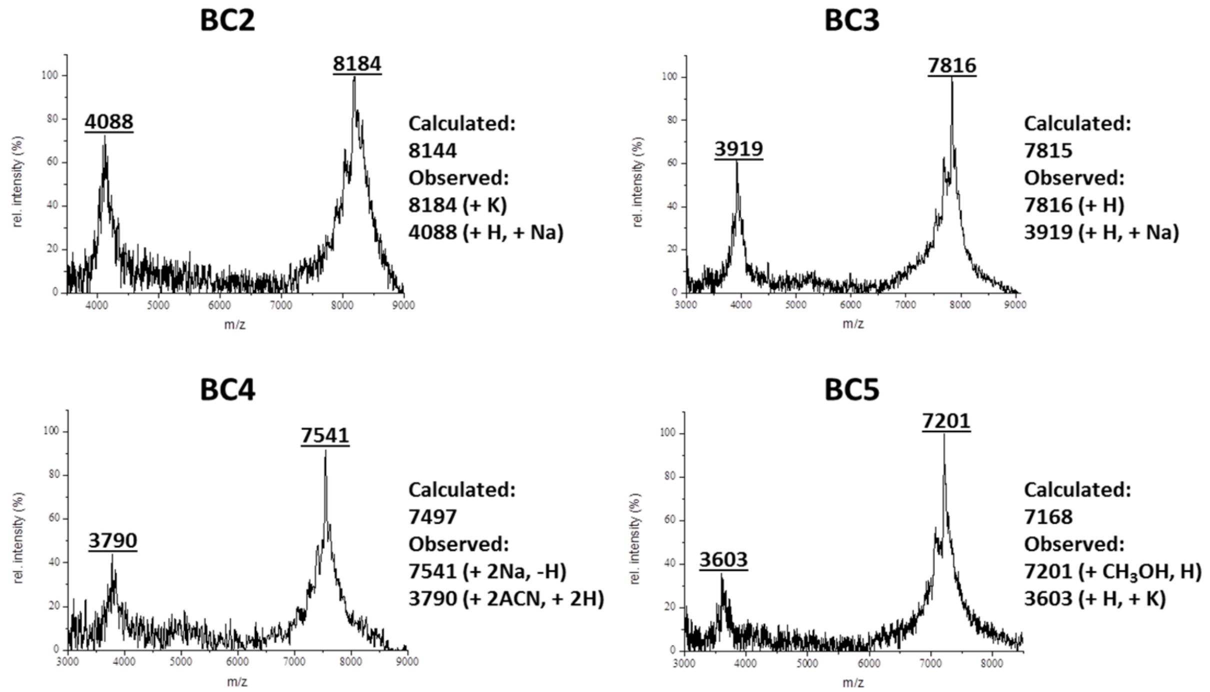

Figure S9. MALDI-TOF spectra of peptidyl-oligonucleotide conjugates BC2 - BC5. Spectra were recorded using a Bruker Daltonics Ultraflex TOF/TOF mass spectrometer.

10. Figure S10. ^{1}H NMR spectra of the bulge-loop inducing conjugates BC2-BC5

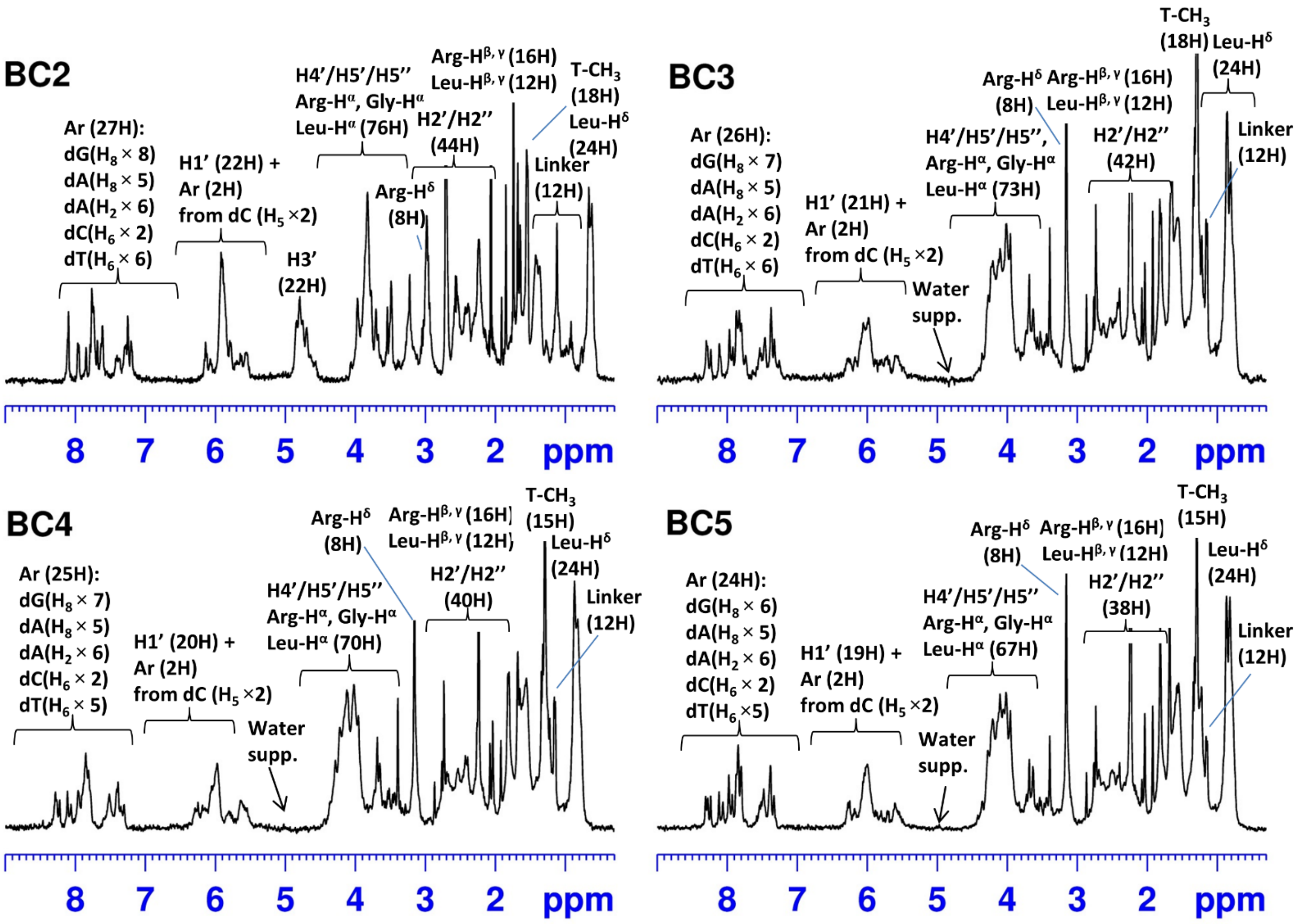

Figure S10. ^{1}H-NMR spectra of bulge conjugates BC2 – BC5 showing the characteristic resonance areas of the oligonucleotide protons, peptide protons, amino-hexyl linker and acetyl protecting group. Assignments of the key ^{1}H resonance regions along with the integral intensities for certain protons are indicated above each spectrum. Analysis of the H3' region was not carried out due to influence of water suppression (indicated as **'Water sup.'**) at 4.78 ppm. The spectra were recorded in D_2O at 25 °C using a 400 MHz NMR spectrometer (Bruker Avance II+ 400).

11. Figure S11. MALDI-ToF mass spectra of the peptidyl-oligonucleotide conjugates BC-α and BC-β

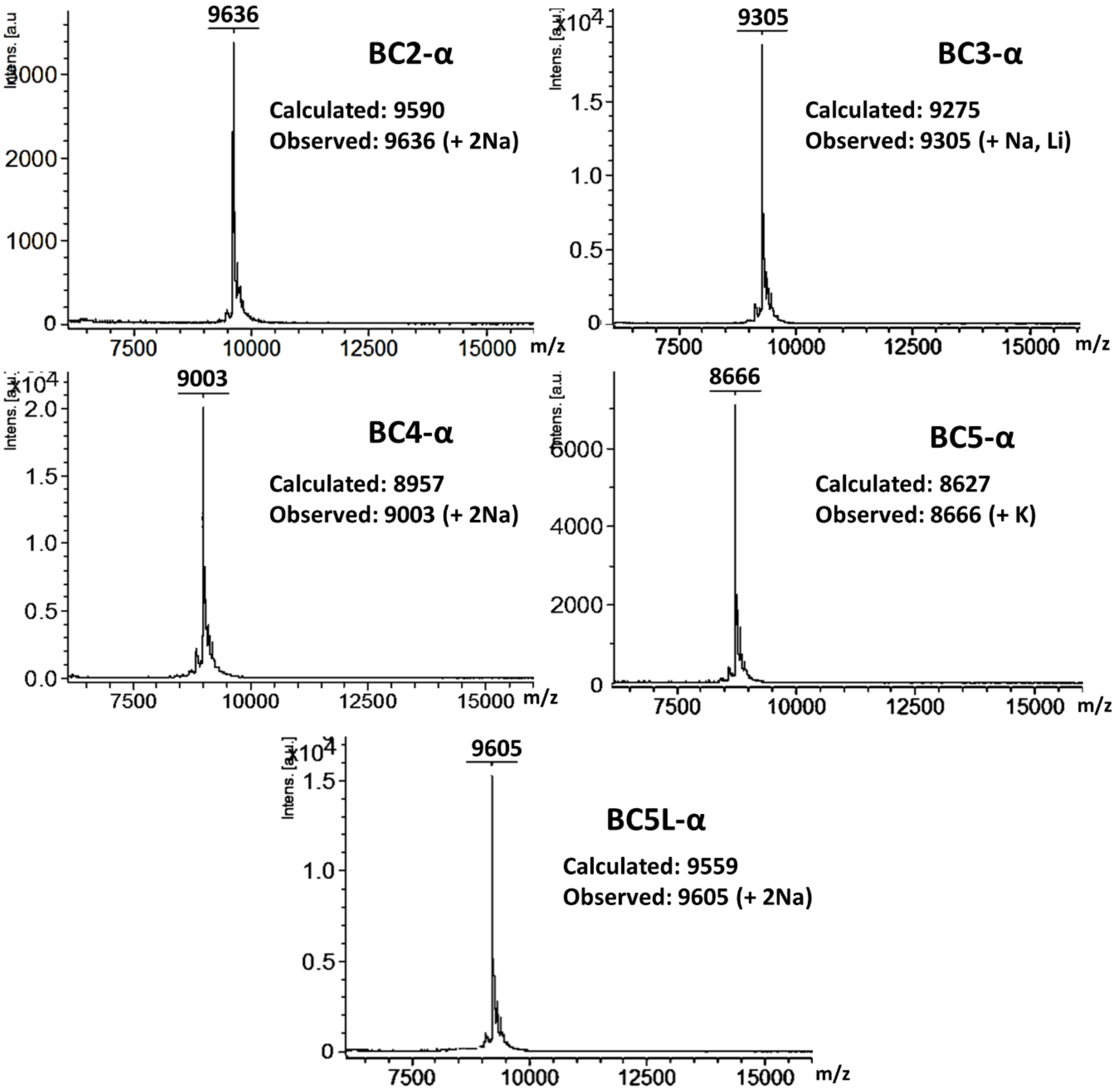

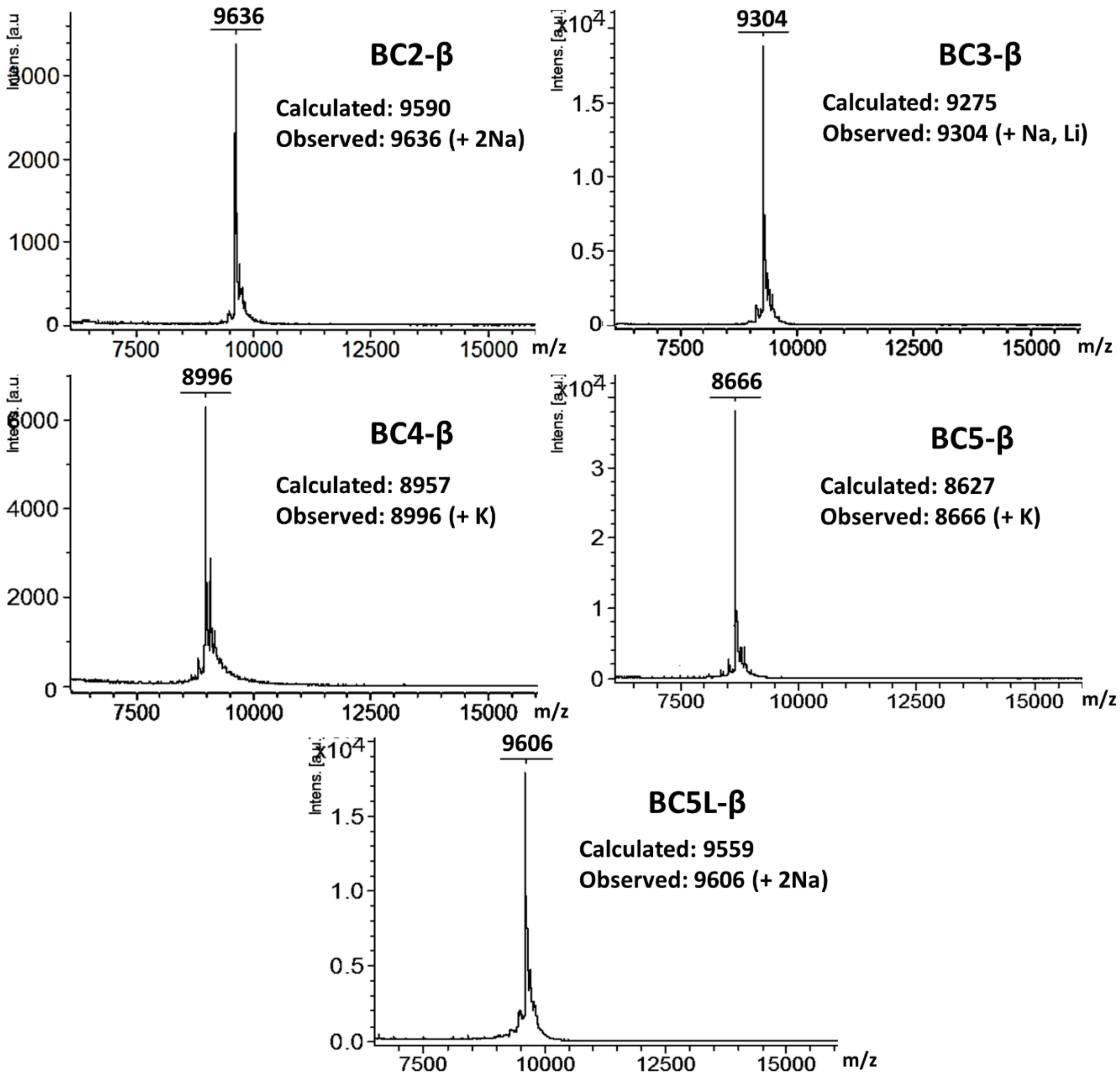

Figure S11. MALDI-ToF spectra of peptidyl-oligonucleotide conjugates BC-α and BC-β. Spectra were recorded using 0.7 M 3-Hydroxy picolinic acid matrix (97 mg/mL, with 0.07 M ammonium citrate, 16 mg/mL in 50:50 ACN:H_2O) on a Bruker Daltonics Ultraflex ToF/ToF mass spectrometer.

12. Figure S12. ^{1}H NMR spectra of the bulge-loop inducing conjugates BC-α and BC-β

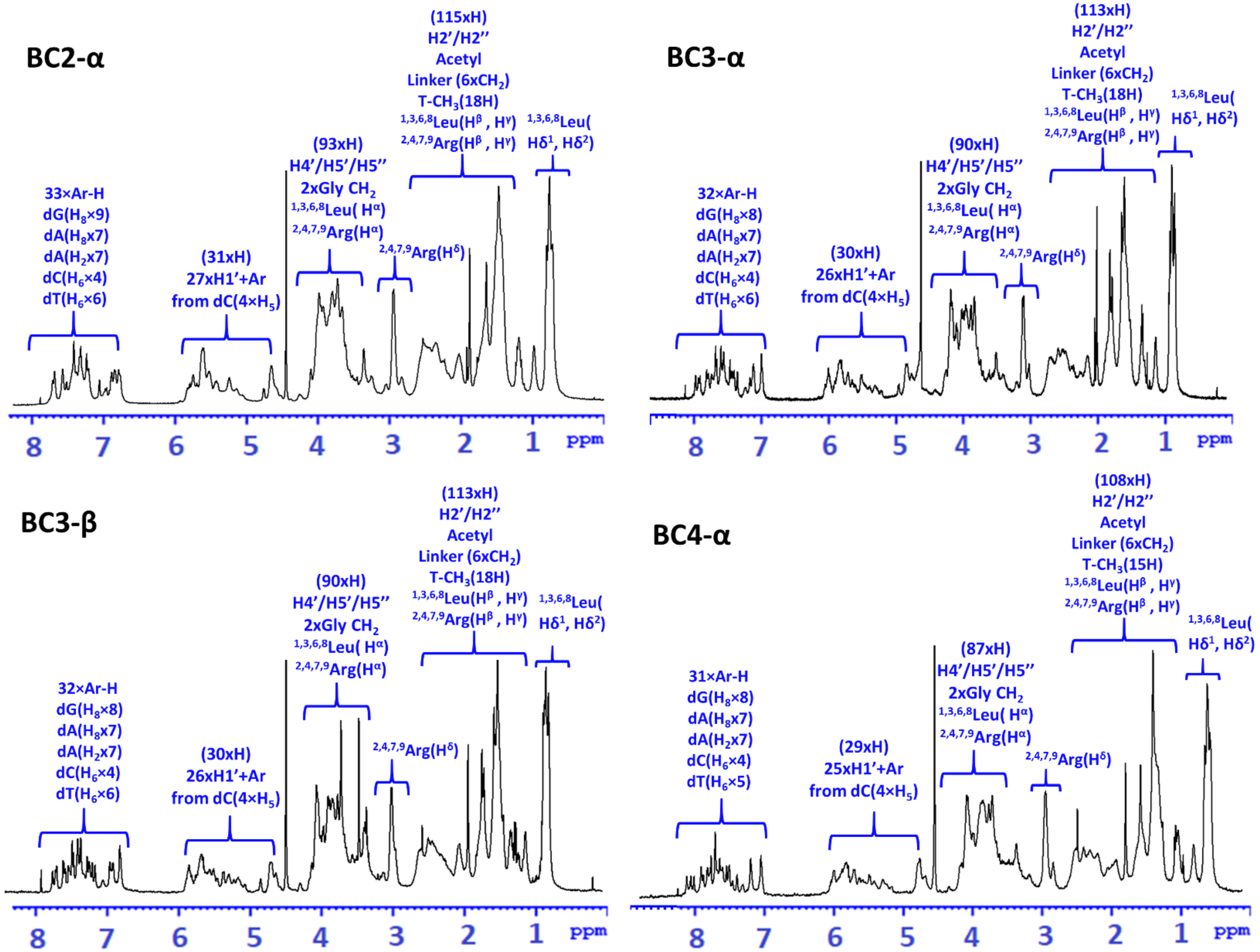

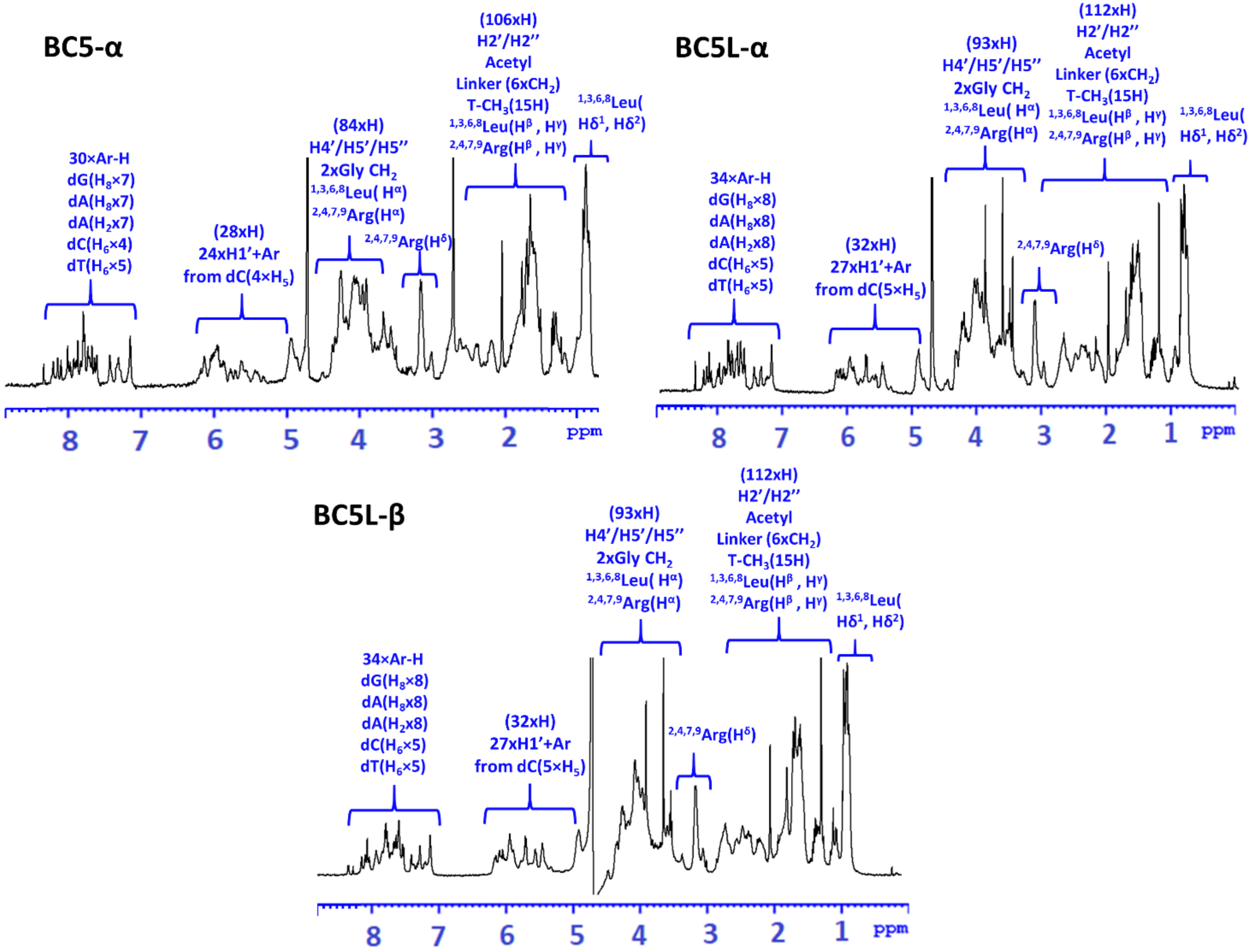

Figure S12. ^1H NMR spectra (400 MHz, Bruker Avance IIþ 400) of conjugates, BC2-α to BC5L-α along with BC3-β and BC5L-β single conjugates indicating prominent chemical shift of protons from oligonucleotides, peptide, aminohexyl linker and acetyl protecting group and the similarities between α and β conjugates. In each spectrum, the breakdown of proton assignment for each region as well as integral intensity has been indicated. H3' region has not been assigned since water suppression prohibits full assignment.

13. Figure S13. Cleavage of 3'-FITC-tRNA^{Phe} with Type 1 conjugates (BC2-BC5)

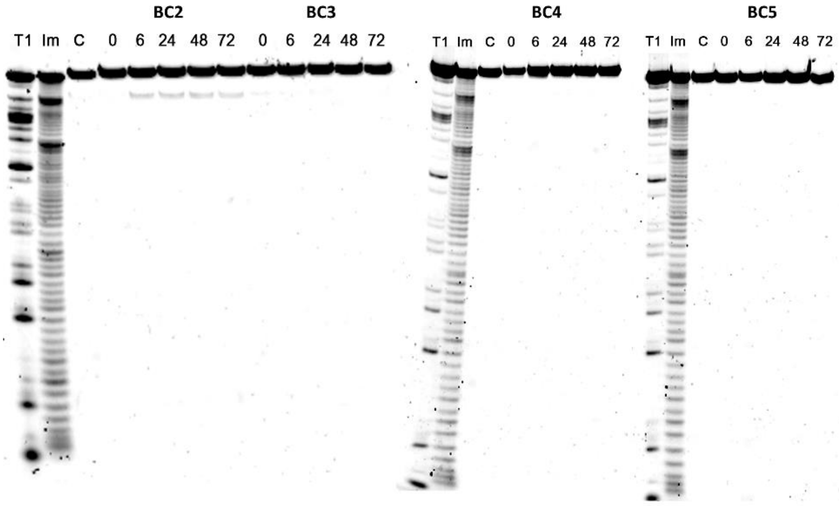

Figure S13. Cleavage of 3'-FITC-tRNA^{Phe} with Type 1 conjugates BC2-BC5. Representative images of 12% PAAM/8 M urea gel after electrophoresis of 3'-FITC-tRNA^{Phe} after incubation with Type 1 conjugates. 3'-FITC-tRNA^{Phe} (1 µM) was incubated with the conjugates (20 µM) in Tris buffer (50 mM Tris-HCl pH 7.0, 0.2 M KCl, 1 mM EDTA) at 37° C. Lanes T1 and Im represent partial 3'-FITC- tRNA^{Phe} digestion with RNase T1 and imidazole, respectively. In lane C, 3'-FITC- tRNA^{Phe} was incubated without conjugates for 24 h. All Type 1 conjugates were shown to be catalytically inactive.

14. Figure S14. RNase H cleavage assay

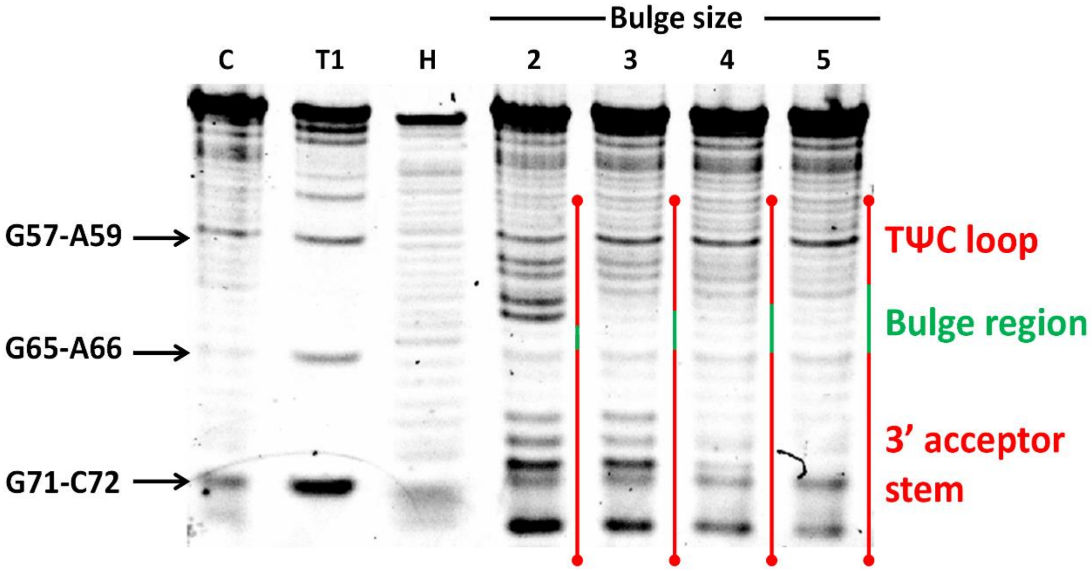

Figure S14. Ribonuclease H cleavage assay showing enhanced cleavage of 3'-FITC-tRNA^{Phe} when it is hybridised within the BC: 3'-FITC-tRNA^{Phe} heteroduplex (indicated by red lines). Absence of cleavage or reduced cleavage in bulge regions (green) is indicative of the presence of a single-stranded RNA bulge, which is not recognised and thus not cleaved by RNase H. 3'-FITC-tRNA^{Phe} (1 µM) was pre-incubated with BC2, BC3, BC4 or BC5 (40 µM) at 37°C for 30 minutes followed by incubation with RNase H (1U) for additional 15 minutes (lanes **2**, **3**, **4** and **5**, respectively). Lanes **T1** and **H**, partial RNA digestion with RNase T1 and hydrolysis ladder, respectively. Lane **C**, control lane containing tRNA^{Phe} in the absence of BCs. RNA cleavage products were resolved in 12% polyacrylamide/8 M urea gel as described in the experimental section.

15. **Figure S15. Distance distributions between guanidinium group of arginines and O2' or phosphorous atoms of the bulged nucleotides of RNA-conjugate duplex.**

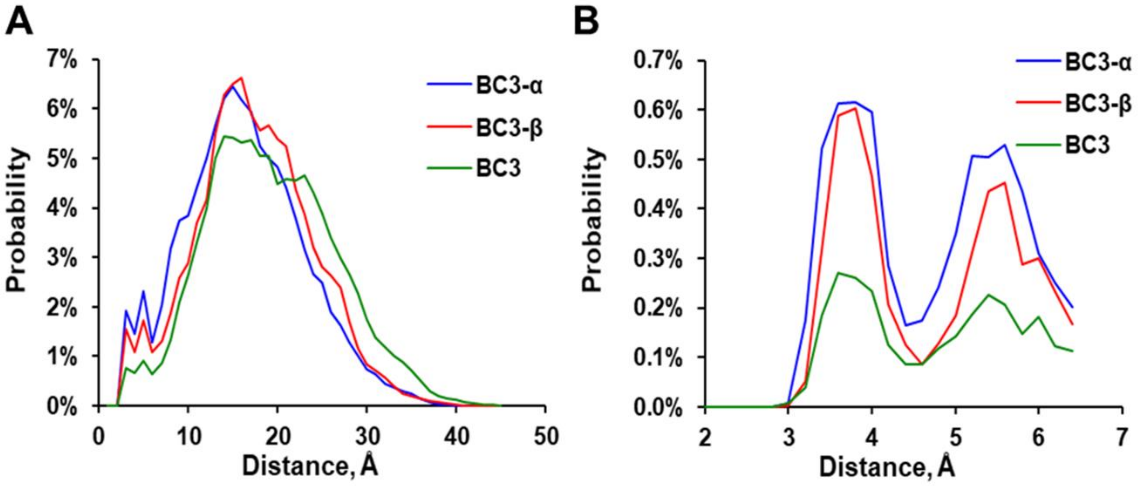

Figure S15. The long-range (**A**) and short-range (**B**) distance distributions between the atoms involved in RNA transesterification catalysis, measured for the hybrid complexes between RNA and BC3 (green), BC3-α (blue) and BC3-β (red). The values of all interatomic distances, including those between the two nitrogen atoms of the guanidinium groups from four arginines and O2' or phosporous atoms of the bulged nucleotide residues from the 3-nt bulge-loop regions, were averaged and normalised to the overall number of frames.

16. **ZIP files**

Clusters.zip: Rotating images of the structural clusters of:

(a) Type 1 conjugates (**BC3.mp4**)

(b) Type 2 conjugates, BC3-alfa anomer (**BC3-alfa.mp4**)

(c) Type 2 conjugates, BC3-beta anomer (**BC3-beta.mp4**)

In-Line Structures.zip:

(1) Rotating images of the representative *"in-line"* conformations of BC3-alfa and BC3-beta anomers:

(a) BC3- alfa_in-line_OVERALL.mp4

(b) BC3-alfa_in-line_ZOOMED.mp4

(c) BC3- beta_in-line_OVERALL.mp4

(d) BC3-beta_in-line_ZOOMED.mp4

BC3-alfa_in-line_OVERALL.mp4 and **BC3-alfa_in-line_OVERALL.mp4** show the whole structures of the calculated complexes obtained from MD simulations. **BC3-alfa_in-line_ZOOMED.mp4** and **BC3-beta_in-line_ZOOMED.mp4** show only the main players involved in the *"in-line attack"*, including two arginines of the catalytic peptide and the key residues of the RNA cleavage site (*i.e.* cytidine residue including the aromatic ring, ribose and phosphate group).

(2) PDB files of the hybridised complexes between RNA and BC3-alfa and BC3-alfa anomers:

(e) BC3-alfa.PDB

(f) BC3-beta.PDB
